# Transcriptomic analysis of diabetic kidney disease and neuropathy in mouse models of type 1 and type 2 diabetes

**DOI:** 10.1242/dmm.050080

**Published:** 2023-10-04

**Authors:** Sarah E. Elzinga, Stephanie A. Eid, Brett A. McGregor, Dae-Gyu Jang, Lucy M. Hinder, Jacqueline R. Dauch, John M. Hayes, Hongyu Zhang, Kai Guo, Subramaniam Pennathur, Matthias Kretzler, Frank C. Brosius, Emily J. Koubek, Eva L. Feldman, Junguk Hur

**Affiliations:** ^1^ Department of Neurology, University of Michigan, Ann Arbor, MI 48109, USA; ^2^ Department of Biomedical Sciences, University of North Dakota School of Medicine and Health Sciences, Grand Forks, ND 58202, USA; ^3^ Division of Nephrology, Department of Internal Medicine, University of Michigan, Ann Arbor, MI 48109, USA; ^4^ Department of Molecular and Integrative Physiology, University of Michigan, Ann Arbor, MI 48109, USA; ^5^ Department of Computational Medicine and Bioinformatics, University of Michigan, Ann Arbor, MI 48109, USA; ^6^ Department of Medicine, University of Arizona, Tucson, AZ 85721, USA

**Keywords:** Diabetes, Diabetic kidney disease, Diabetic peripheral neuropathy, RNA-seq, Self-organizing map, Genetic mouse models

## Abstract

Diabetic kidney disease (DKD) and diabetic peripheral neuropathy (DPN) are common complications of type 1 (T1D) and type 2 (T2D) diabetes. However, the mechanisms underlying pathogenesis of these complications are unclear. In this study, we optimized a streptozotocin-induced *db/+* murine model of T1D and compared it to our established *db/db* T2D mouse model of the same C57BLKS/J background. Glomeruli and sciatic nerve transcriptomic data from T1D and T2D mice were analyzed by self-organizing map and differential gene expression analysis. Consistent with prior literature, pathways related to immune function and inflammation were dysregulated in both complications in T1D and T2D mice. Gene-level analysis identified a high degree of concordance in shared differentially expressed genes (DEGs) in both complications and across diabetes type when using mice from the same cohort and genetic background. As we have previously shown a low concordance of shared DEGs in DPN when using mice from different cohorts and genetic backgrounds, this suggests that genetic background may influence diabetic complications. Collectively, these findings support the role of inflammation and indicate that genetic background is important in complications of both T1D and T2D.

## Teaser

**Summary:** Transcriptomic profiles from glomeruli and sciatic nerves of type 1 and type 2 diabetes mouse models support a strong influence of inflammation and genetic background on diabetic complications.

## INTRODUCTION

Diabetes is a major world-wide health issue that impacts 1 in 10 adults (Sun et al., 2022). Type 1 diabetes mellitus (T1D) is a chronic auto-immune disease that impairs insulin production. Type 2 diabetes mellitus (T2D), the more prevalent diabetes type (Tan et al., 2019), is characterized by insulin resistance and frequently associates with obesity. Diabetic kidney disease (DKD) and diabetic peripheral neuropathy (DPN) are the most common microvascular complications of T1D and T2D and carry major health and economic costs globally (Chawla et al., 2016; Stehouwer, 2018). DKD, characterized by loss of kidney function with increased fibrosis, affects 25-40% of diabetic patients and is a leading cause of end-stage renal disease (Afkarian et al., 2013; Palsson and Patel, 2014; Saran et al., 2018; Tuttle et al., 2014). Effective interventions for DKD have advanced in recent years and include RAS blockade and SGLT2 inhibition (Warren et al., 2019). Even more prevalent in diabetic populations is DPN, a chronic degeneration of the peripheral nerves that progresses in a distal-to-proximal fashion (Elafros et al., 2022). Loss of sensation in DPN increases the risk of foot infection and ulcers and can, ultimately, lead to lower-limb amputation (Juster-Switlyk and Smith, 2016; Singh et al., 2014). Unfortunately, aside from tight glucose control, there are currently no effective treatments for DPN (Callaghan et al., 2012; Leoncini et al., 2020; Pop-Busui et al., 2017).

The lack of effective therapies is partially due to a poor understanding of the underlying disease mechanisms. Studies of DKD and DPN suggest a role of genetic susceptibility in the development of diabetic complications (Gu, 2019; Sandholm and Groop, 2018; Vujkovic et al., 2020). Murine models have been invaluable for addressing these issues and understanding the mechanistic pathogenesis of these complications (Biessels et al., 2014; Eid and Feldman, 2021; Lim et al., 2014; O'Brien et al., 2014). However, direct comparisons of complications or diabetes type, particularly within the same model system, are rare. A common murine model of T1D is induced by streptozotocin (STZ) injection (O'Brien et al., 2014). STZ induces robust DKD and DPN phenotypes (Christianson et al., 2007; O'Brien et al., 2014). However, phenotypes can vary based on model design, site, injection method and mouse strain (O'Brien et al., 2014; Saadane et al., 2020; Ventura-Sobrevilla et al., 2011). Leptin receptor-deficient C57BLKS/J-*db/db* mice are considered to be one of the best-characterized T2D mouse models (Eid and Feldman, 2021; Kitada et al., 2016; Tesch and Lim, 2011) that also produce robust DKD and DPN phenotypes (Kitada et al., 2016; Liu et al., 2017; O'Brien et al., 2018; Tesch and Lim, 2011).

We have previously collected gene expression data by using glomeruli (Glom) and sciatic nerve (SCN) from STZ-induced T1D and *db/db* T2D mice on different genetic backgrounds (Hur et al., 2016). In that study, we showed similar mechanisms of kidney damage but a high degree of discordance in shared SCN DEGs when comparing T1D and T2D. We speculated this was due to the use of different cohorts and genetic backgrounds (Hur et al., 2016). Therefore, the overarching goal of our current study was to identify shared and distinct pathways and mechanisms underlying DKD and DPN in T1D and T2D mouse models on the same genetic background. To do so, we first optimized an STZ-induced murine model of T1D. We then compared metabolic, DKD and DPN phenotypes of this T1D murine model with those of our previously published *db/db* T2D cohort (Hinder et al., 2017b). Finally, we compared gene expression in Glom and SCN tissue from STZ-induced C57BLKS/J-*db/+* T1D, C57BLKS/J-*db/db* T2D and C57BLKS/J-*db/+* control animals. Altered molecular pathways of interest were identified using self-organizing map (SOM) and differentially expressed gene (DEG) analyses to determine common and distinct patterns between tissues and across diabetes type.

We found a more robust complication phenotype upon a single high dose (SHD) of STZ, which was selected for further study. Both *db/+* STZ T1D and *db/db* T2D mouse models developed metabolic, DKD and DPN phenotypes. Across diabetes type, dysregulation of immune and inflammatory pathways was observed for both DKD and DPN. Interestingly, on the same genetic background, we identified a high degree of concordance in shared DEGs between T1D and T2D, in both Glom and SCN. Overall, our results support the importance of inflammation and genetic background in the pathogenesis of these common diabetic complications.

## RESULTS

### Mice induced with a SHD of STZ mimic the human T1D DPN phenotype

To compare T1D and T2D mouse models on the same genetic background, we first optimized an STZ-induced T1D model on a C57BLKS/J background, i.e. the same genetic background of our T2D *db/db* mouse model. We applied two common STZ dosing paradigms: an SHD of STZ (150 mg kg^−1^ STZ at 5 weeks of age, *db/+* SHD-STZ) or multiple low doses (MLD) of STZ (50 mg kg^−1^ STZ for 5 days starting at 5 weeks of age, *db/+* MLD-STZ). Metabolic and DPN phenotyping were performed to identify the STZ-induced T1D model that most closely mimicked human T1D DPN.

Starting at 7 weeks and continuing throughout the course of the study, SHD-STZ and MLD-STZ animals weighed significantly less than *db/+* control (Ctrl) mice (Fig. S1A). However, beginning at week 11, MLD-STZ mice were heavier than SHD-STZ mice. Fasting blood glucose (FBG) levels were similar in SHD-STZ and MLD-STZ mice but elevated in both STZ-induced mouse models compared to *db/+* Ctrl mice (Fig. S1B), indicating impaired glycemic control. As anticipated, levels of terminal plasma insulin at 16 weeks were significantly lower in STZ animals than in *db/+* Ctrl mice, regardless of the STZ dose (Fig. S2A). Terminal plasma glycated hemoglobin (GHb) concentrations were significantly higher in SHD-STZ and MLD-STZ mice versus *db/+* Ctrl mice, and in SHD-STZ versus MLD-STZ mice (Fig. S2B). Thus, both SHD-STZ and MLD-STZ mice developed an impaired metabolic phenotype that is similar to human T1D.

We also performed DPN phenotyping to identify differences in DPN development in T1D mouse models induced with a SHD or MLD of STZ. At week 16, SHD-STZ and MLD-STZ mice displayed significantly longer-lasting hind paw withdrawal latency compared to *db/+* Ctrl mice, indicating thermal hypoalgesia and sensory loss. Between the STZ groups, hind paw withdrawal thresholds lasted significantly longer in SHD-STZ versus MLD-STZ mice (Fig. S3A). Nerve conduction velocities (NCVs) – i.e. measurements of large fiber function – of sural nerves and SCNs were assessed at 16 weeks. SHD-STZ and MLD-STZ animals displayed significantly impaired sural and sciatic NCVs compared to those of *db/+* Ctrl mice (Fig. S3B,C). However, SHD-STZ mice had significantly worse sural and sciatic NCVs compared to those of MLD-STZ mice (Fig. S3B,C). We also measured changes in intraepidermal nerve fiber densities (IENFDs) in the footpad of the mouse hind paw to quantify loss of small nerve fibers. IENFDs were significantly reduced in SHD-STZ mice compared to MLD-STZ and *db/+* Ctrl mice (Fig. S3D). These results indicate that an SHD of STZ induced a more severe DPN phenotype than MLD of STZ.

In summary, both SHD-STZ and MLD-STZ administration produced a clear metabolic and DPN phenotype. However, the SHD-STZ-induced DPN phenotype was more severe than the MLD-STZ-induced DPN phenotype. Therefore, SHD-STZ was used in subsequent experiments to induce a more biologically relevant T1D mouse model that, hereafter, is referred to as *db/*+ STZ T1D.

### Metabolic, DKD and DPN phenotyping of T1D and T2D mice

To verify that our *db/+* STZ T1D and *db/db* T2D mouse models develop long-term metabolic disturbances, DKD and DPN, and to examine differences in the severity of complications, we compared metabolic and complication phenotyping at 16 weeks across diabetes type (Figs S4-S6). Of note, metabolic, DKD and DPN phenotyping of *db/+* Ctrl and *db/db* mice has previously been published by Hinder et al. (2017b).

By week 16, metabolic phenotyping indicated that both diabetes models displayed significantly impaired metabolism. In terms of weight, *db/+* STZ animals weighed significantly less than *db/+* Ctrl mice, whereas *db/db* mice were heavier than *db/+* STZ or *db/+* Ctrl mice (Fig. S4A). FBG levels were similar in *db/+* STZ and *db/db* mice but significantly higher than those in *db/+* Ctrl animals (Fig. S4B). Plasma insulin concentrations did not differ significantly between groups (Fig. S4C). Plasma GHb concentrations did not differ significantly between *db/+* STZ and *db/db* mice. However, GHb concentrations were significantly elevated in *db/+* STZ and *db/db* animals compared to those in *db/+* Ctrl animals (Fig. S4D). In summary, we found that both *db/+* STZ T1D and *db/db* T2D mouse models develop a similar degree of hyperglycemia relative to that in control animals.

To investigate DKD development and progression, mesangial expansion, mesangial index, glomerular area and urinary albumin-to-creatinine ratio (ACR) were measured at study termination for both diabetic mouse models. Mesangial expansion, as indicated by glomerular areas positive for periodic acid-Schiff (PAS) staining, was significantly increased in *db/+* STZ and *db/db* versus *db/+* Ctrl mice, and in *db/db* versus *db/+* STZ animals (Fig. S5A). Similarly, the mesangial index and glomerular area were significantly greater in *db/+* STZ and *db/db* mice compared with those in *db/+* Ctrl animals, and greater in *db/db* mice compared with those in *db/+* STZ animals (Fig. S5B,C). Urinary ACR was significantly elevated in both *db/+* STZ and *db/db* groups compared to that in *db/+* Ctrl mice, but did not differ between *db/+* STZ and *db/db* animals (Fig. S5D). These results indicate that both *db/+* STZ T1D and *db/db* T2D mouse models develop DKD, but that the phenotype is more robust in *db/db* mice.

When DPN was assessed at week 16, hind paw withdrawal latencies were not significantly different in *db/+* STZ compared to *db/+* Ctrl mice. However, *db/db* mice displayed hind paw withdrawal latencies lasting significantly longer than those in *db/+* Ctrl mice, indicating thermal hypoalgesia (Fig. S6A). For NCV measurements, sciatic and sural NCVs were significantly reduced in *db/+* STZ and *db/db* versus *db/+* Ctrl mice (Fig. S6B,C). IENFD measurements were significantly reduced in *db/+* STZ mice compared to those in *db/+* Ctrl but did not differ between *db/db* and *db/+* Ctrl mice (Fig. S6D). Collectively, these data suggest that both *db/+* STZ T1D and *db/db* T2D mouse models develop DPN to a similar degree.

### Tissue-specific gene expression

**Fig. 1.**
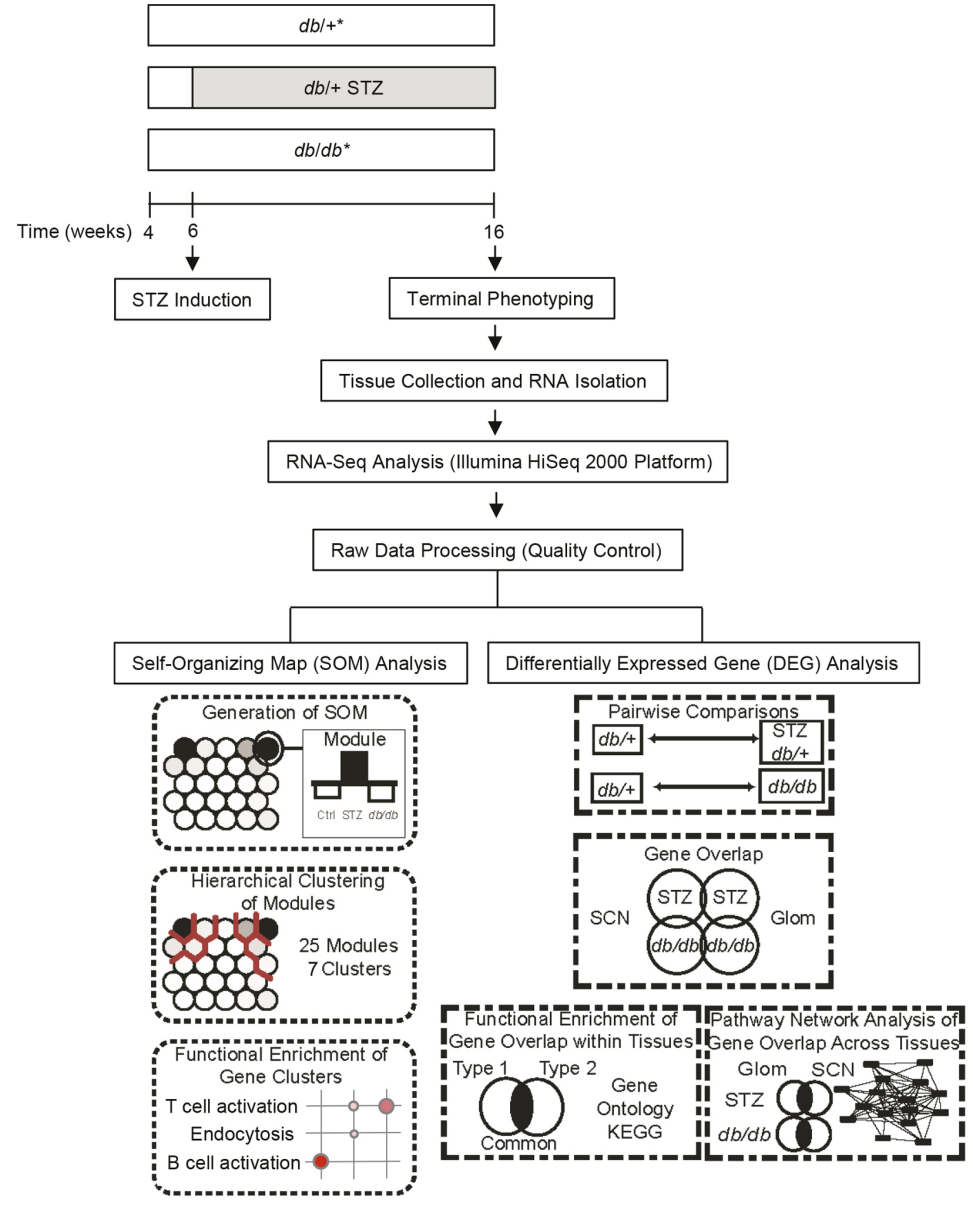
**Experimental workflow.** Control (*db/+*), T1D [*db/+* STZ (SHD)] and T2D (*db/db*) mice underwent phenotypic assessment at 16 weeks of age. Mice (*n*=6/group) were euthanized at 16 weeks and glomeruli (Glom) and sciatic nerves (SCN) were collected. Total RNA was collected from each tissue type and gene expression was assessed using RNA sequencing (RNA-Seq) analysis, processed and quality checked. Processed data were then analyzed in parallel using self-organizing map (SOM) and differentially expressed gene (DEG) analyses. Gene Ontology and Kyoto Encyclopedia of Genes and Genomes (KEGG) analyses identified functional enrichment and canonical molecular pathways that are significantly associated with altered gene expression. Finally, pathway network analysis of the common DEGs in each diabetes type was performed to identify common pathways across complications. STZ, streptozotocin; SHD, single high dose.

Having established that *db/+* STZ T1D and *db/db* T2D mice on the same genetic background develop DKD and DPN, we next assessed gene expression changes in Glom and SCN to identify crucial pathways involved in the development of these complications (Fig. 1). Gene expression was measured by RNA sequencing, and pathways of interest were identified by parallel SOM and DEG analyses. Both tissues were mapped to the mouse reference genome (mm10; GRCm38) at an average mapping rate of 77% with, on average, 23.0 million reads for Glom and 22.2 million reads for SCN samples.

### SOM analysis

**Fig. 2.**
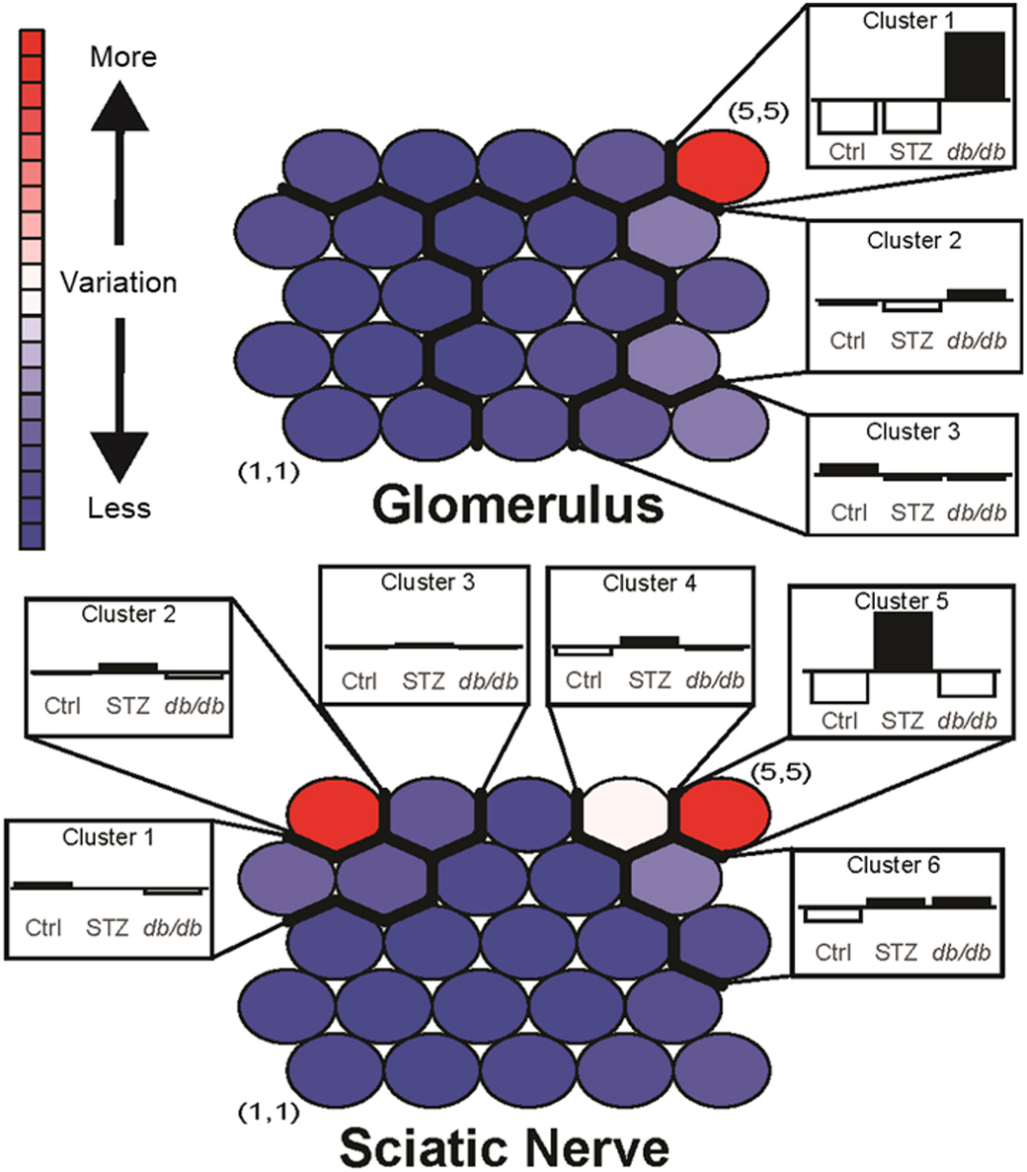
**SOM analysis.** Self-organizing map (SOM) analysis was used to identify coherent gene expression patterns in glomerulus and sciatic nerve. Genes with similar expression patterns were sorted into modules (indicated by circles) on 5×5 maps. The color range (blue to red) indicates the level of variation between groups in expression within each module, ranging from small changes (blue) to large changes (red). Hierarchical clustering of groups was performed to identify and group modules into clusters of similar kinetic patterns between groups – i.e. Ctrl (control *db*/+), STZ (streptozotocin T1D model, single high dose) and *db/db* (T2D model) – and is indicated by modules within thick black lines.

SOM analysis was performed to identify shared and distinct patterns and associated pathways across tissue and diabetes type. SOM is an unsupervised clustering method that organizes genes with similar expression patterns into modules in an unbiased manner. For each tissue, genes were organized into a SOM map of modules, each of which represent a unique pattern of gene expression compared with *db/+* control mice (Fig. 2). Hierarchical clustering was further employed to group adjacent modules that comprise similar gene expression patterns into clusters. These clusters were then analyzed for functional enrichment using Gene Ontology (GO) enrichment analysis and Kyoto Encyclopedia of Genes and Genomes (KEGG) mapping to identify molecular pathways that are significantly altered in *db/+* STZ and *db/db* versus *db/+* Ctrl mice.

**Fig. 3.**
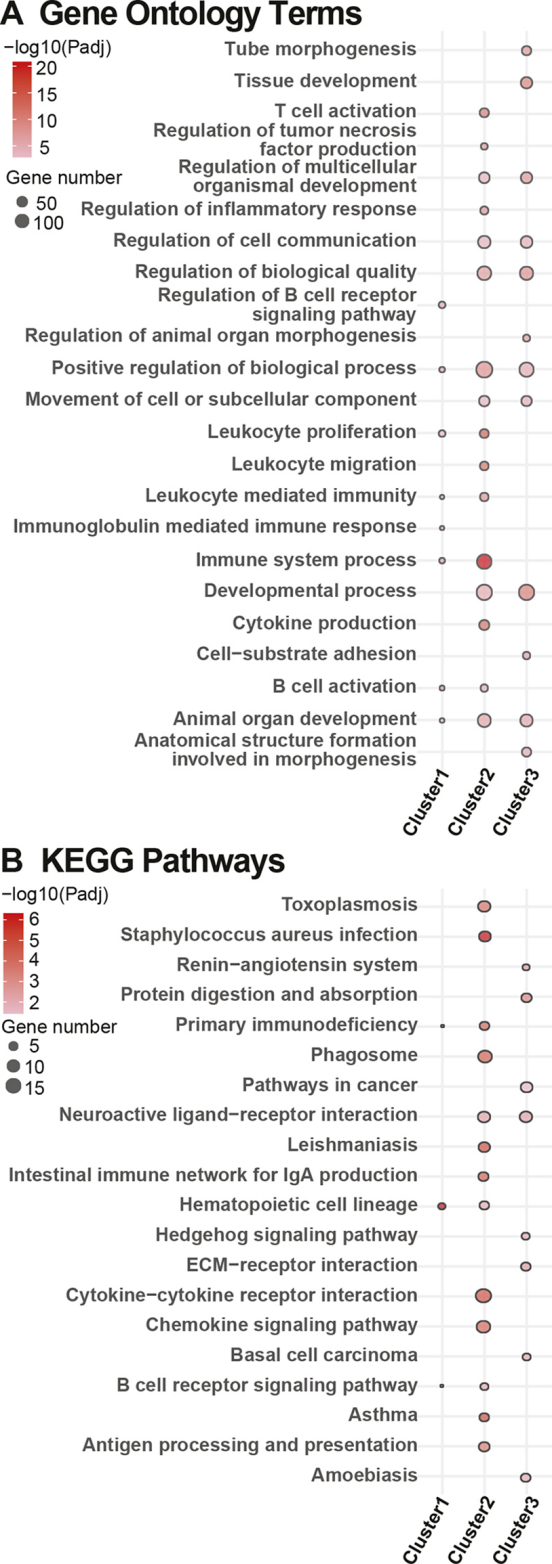
**Pathway enrichment analysis of Glom clusters obtained using SOM analysis.** (A,B) Dot plots highlighting the top ten most significantly enriched Gene Ontology terms (A) and Kyoto Encyclopedia of Genes and Genomes (KEGG) pathways (B) in each of the three/six module clusters obtained using self-organizing map (SOM) analysis of glomeruli (Glom)/sciatic nerve (SCN), as determined by richR. Note that a term might not be in the top ten of every set but is included if it appears in the top ten of any given set. Additionally, more than ten terms may appear for a single set if that set contains a term (outside of its own top ten) that was within the top ten of a separate set. The dot size represents the number of genes in the cluster within each term/pathway. The dot color intensity indicates significance levels (Benjamini–Hochberg procedure-adjusted *P*-value) shown on a −log10 scale.

Within Glom, we identified three clusters (Fig. 3A) comprising the highest degree of standard deviation (s.d.) between groups for subsequent enrichment analysis (Tables S1-S4). In GO enrichment analysis (Fig. 3A), ‘B cell activation’ was the most significant in Cluster 1. In KEGG analysis (Fig. 3B), ‘staphylococcus aureus infection’ and ‘asthma’ were the most significant in Cluster 2. However, overall, Clusters 1 and 2 were associated with pathways related to the immune system, including leukocytes and chemokines/cytokines (Tables S5-S8) together with developmental and regulatory processes. Immune pathways were downregulated in *db/+* STZ but upregulated in *db/db* mice (Tables S2, S3). The chemokine ligand 12-encoding gene *Ccl12* was identified as a gene of interest within these pathways. In contrast, pathways in Cluster 3 were downregulated in both *db/+* STZ and *db/db* animals (Table S4). Pathways that were significantly downregulated in both diabetes types included those related to the renin-angiotensin system (RAS) and the extracellular matrix (Tables S9, S10). A gene of interest within these pathways was the matrix metalloproteinase-9-encoding *Mmp9*.

**Fig. 4.**
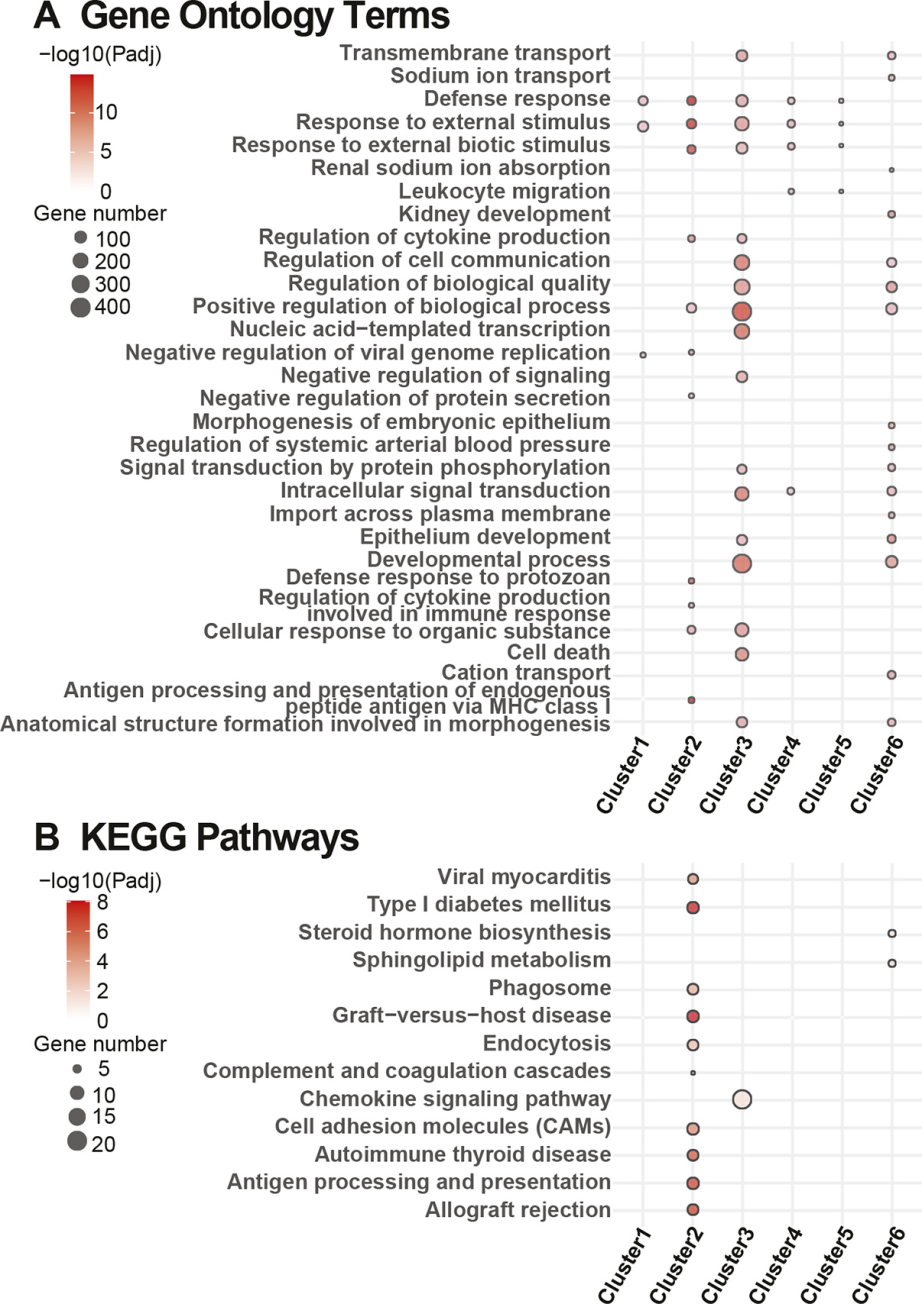
**Pathway enrichment analysis of SCN clusters obtained using SOM analysis.** (A,B) Dot plots highlighting the top ten most significantly enriched Gene Ontology terms (A) and Kyoto Encyclopedia of Genes and Genomes (KEGG) pathways (B) in each of the three/six module clusters obtained using self-organizing map (SOM) analysis of glomeruli (Glom)/sciatic nerve (SCN), as determined by richR. Note that a term might not be in the top ten of every set but is included if it appears in the top ten of any given set. Additionally, more than ten terms may appear for a single set if that set contains a term (outside of its own top ten) that was within the top ten of a separate set. The dot size represents the number of genes in the cluster within each term/pathway. The dot color intensity indicates significance levels (Benjamini–Hochberg procedure-adjusted *P*-value) shown on a −log10 scale.

In the SCN, six clusters with a high degree of s.d. were selected for pathway enrichment (Tables S11-S26). Clusters 2, 4 and 5 contained pathways that were upregulated in *db/+* STZ but downregulated in *db/db* animals (Tables S13, S15, S16). GO analysis (Fig. 4A) found ‘positive regulation of biological processes’ was the most significant in Cluster 3. KEGG analysis (Fig. 4B) identified ‘graft-versus-host disease’ as the most significant in Cluster 2. However, overall, we determined that pathways associated with immune and defense responses and leukocyte migration were enriched (Tables S19-21, S26), in addition to pathways related to developmental and regulatory processes. *Ccl12* was again identified as a gene of interest within these pathways.

### Differential gene expression analysis

**Fig. 5.**
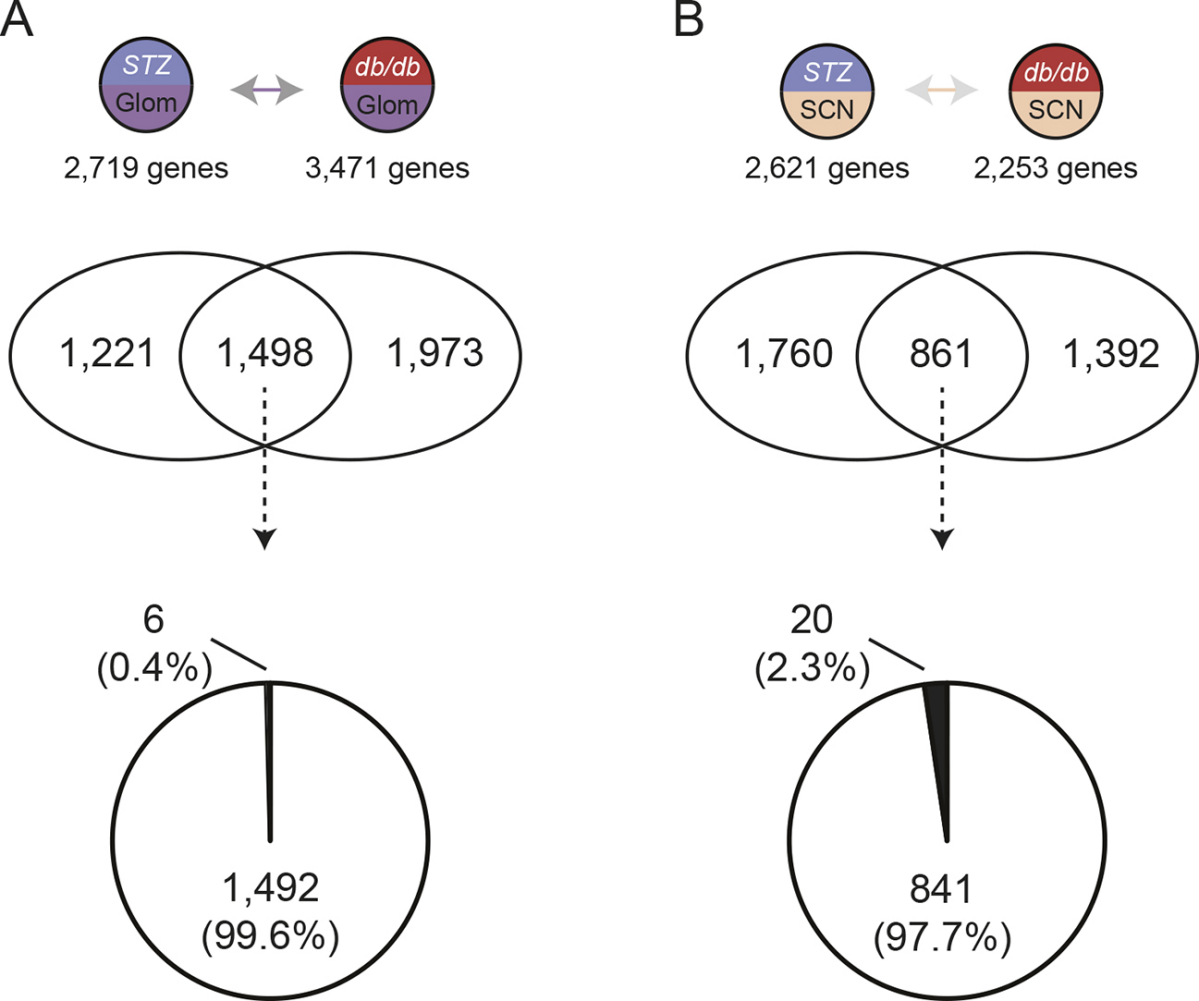
**Comparison of *db/+* STZ and *db/db* DEGs.** Venn diagrams representing differentially expressed genes (DEGs) that are either common or unique to glomeruli (Glom) (A) or sciatic nerve (SCN) (B) of *db/+* STZ (T1D) and *db/db* (T2D) mouse models. The pie charts represent the percentage of shared DEGs comprising concordant (i.e. same direction) or discordant (i.e. opposite direction) gene expression, e.g. upregulation in both T1D and T2D (white) or upregulation in T1D but downregulation in T2D (black).

To complement our SOM analysis, we used DEG analysis to analyze transcriptomic profiles in Glom and SCN of *db/+* STZ and *db/db* mouse models. First, we identified DEGs from *db/+* STZ and *db/db* mice that were upregulated or downregulated compared with *db/+* Ctrl mice (Tables S27-S30). Through DEG analysis, we found 2719 (Glom) and 2621 (SCN) genes that were significantly upregulated or downregulated in *db/+* STZ mice and 3471 (Glom) and 2253 (SCN) DEGs that were significantly upregulated or downregulated in *db/db* animals. To identify common and distinct gene expression patterns in *db/+* STZ and *db/db* animals for DKD and DPN, unique and shared groups of DEGs were examined within these tissue types (Fig. 5; Tables S31-S36). Shared DEGs between *db/+* STZ and *db/db* mice were highly concordant in both tissues, with 99.6% of DEGs in Glom and 97.7% of DEGs in SCN, which indicates the same directional change − i.e. both genes are upregulated or both genes are downregulated − versus *db/+* Ctrl mice.

**Fig. 6.**
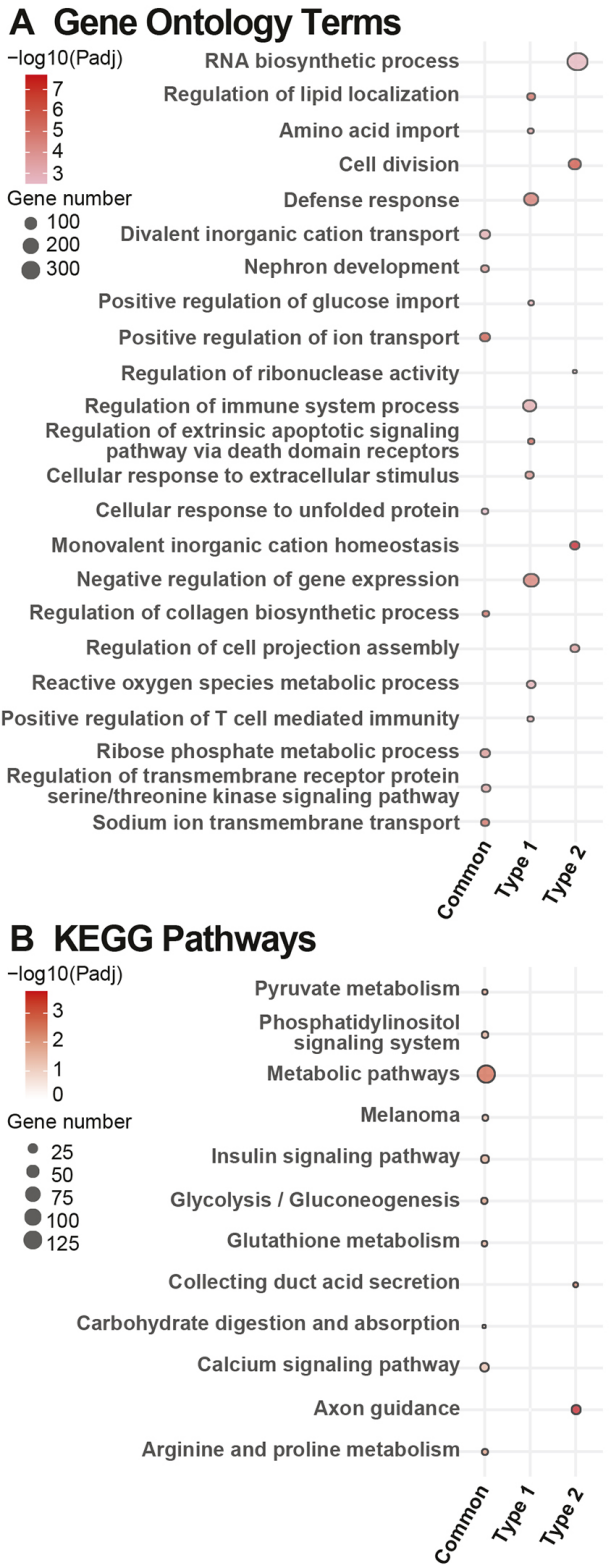
**Pathway enrichment analysis of DEGs identified in glomeruli.** (A,B) Pathway enrichment analysis of differentially expressed genes (DEGs) identified in glomeruli, which are common in both (Common), unique to *db/+* STZ (streptozotocin, T1D) (Type1) or unique to the *db/db* (T2D) (Type 2) mouse model. Dot plots highlighting the top ten most significantly enriched Gene Ontology terms (A) and Kyoto Encyclopedia of Genes and Genomes (KEGG) pathways (B) in each of the three/six module clusters obtained using self-organizing map (SOM) analysis of glomeruli (Glom)/sciatic nerve (SCN), as determined by richR. Note that a term might not be in the top ten of every set but is included if it appears in the top ten of any given set. Additionally, more than ten terms may appear for a single set if that set contains a term (outside of its own top ten) that was within the top ten of a separate set. The dot size represents the number of genes per set within each term/pathway. The dot color intensity indicates significance levels (Benjamini–Hochberg procedure-adjusted *P*-value) shown on a −log10 scale.

GO and KEGG functional enrichment analysis was performed to identify common and distinct biological pathways within the DEGs for both Glom and SCN. For Glom (Fig. 6), functional enrichment analysis revealed that DEGs shared between *db/+* STZ and *db/db* animals are significantly enriched in pathways related to ion transport, nephron development and collagen biosynthesis (GO; Fig. 6A; Table S37), as well as in metabolic pathways, such as insulin signaling and glycolysis/gluconeogenesis (KEGG; Fig. 6B; Table S38). A significant DEG of interest within these pathways was *Akt2* (Table S33).

Representation of DEGs in Glom unique to *db/+* STZ mice was varied, and consisted of pathways related to immune system, localization, metabolic processes and oxidative stress (Table S39). A significant DEG within these pathways was *Cyp4a14* (Table S31). There were no significant KEGG pathways. GO enrichment analysis of DEG sets in Glom specific to *db/db* mice identified pathways related to cell division and genetic regulation (Table S40). There were only two significant KEGG pathways, axon guidance and collecting duct acid secretion (Table S41). DEGs of interest within these pathways were *Ccl12* and another member of the cytochrome P450 family *Cyp1a1* (Table S32). *Ccl12* was also identified as a gene of interest in our SOM analysis of Glom, indicating that it could be a particularly important chemokine ligand in T2D DKD.

**Fig. 7.**
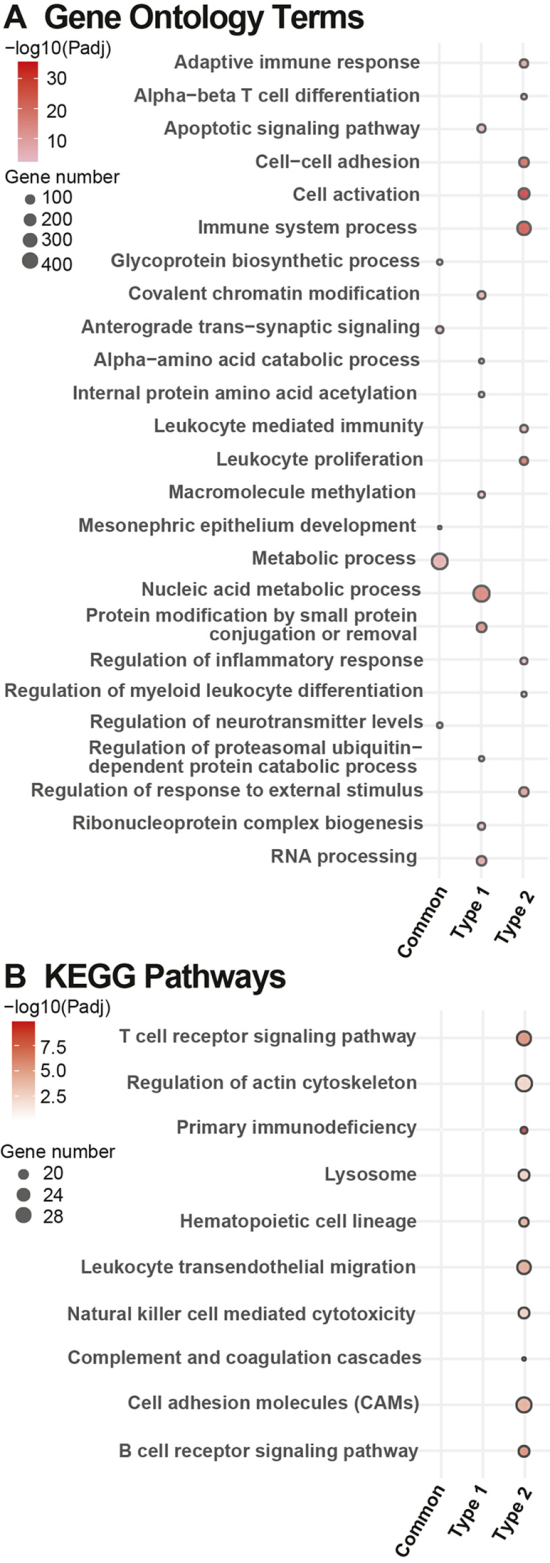
**Pathway enrichment analysis of DEGs identified in the SCN.** (A,B) Pathway enrichment analysis of differentially expressed genes (DEGs) identified in the sciatic nerve (SCN), which are common in both (Common), unique to *db/+* STZ (streptozotocin, T1D) (Type1) or unique to the *db/db* (T2D) (Type 2) mouse model. Dot plots highlighting the top ten most significantly enriched Gene Ontology terms (A) and Kyoto Encyclopedia of Genes and Genomes (KEGG) pathways (B) in each of the three/six module clusters obtained using self-organizing map (SOM) analysis of glomeruli (Glom)/sciatic nerve (SCN), as determined by richR. Note that a term might not be in the top ten of every set but is included if it appears in the top ten of any given set. Additionally, more than ten terms may appear for a single set if that set contains a term (outside of its own top ten) that was within the top ten of a separate set. The dot size represents the number of genes per set within each term/pathway. The dot color intensity indicates significance levels (Benjamini–Hochberg procedure-adjusted *P*-value) shown on a −log10 scale.

In the SCN, functional enrichment analysis of shared DEGs from *db/+* STZ and *db/db* mice (Fig. 7) revealed pathways related to metabolic processes and signal transduction (Fig. 7A; Table S42). GO enrichment analysis of DEGs unique to *db/+* STZ animals identified pathways related to nucleic acid metabolic processes and post-translational modifications (Table S43) as well as pathways related to genetic regulation, such as RNA processing. A DEG of interest within these pathways was *Cyp1a1,* which was also identified as a gene of interest in Glom (Table S34). There were no significant KEGG pathways for shared DEGs or for DEGs unique to *db/+* STZ mice. GO enrichment and KEGG pathway analyses revealed pathways related to immune activation and the immune system to be highly enriched among DEGs unique to the SCN of *db/db* mice (Tables S44, S45). GO enrichment also identified pathways related to transport and localization, while KEGG enrichment highlighted the involvement of cellular adhesion-related pathways (Fig. 7B; Tables S44, S45). Significant DEGs within these pathways included *Mmp9* and *Pla2g2d*, a member of the phospholipase A2 family (Table S35).

**Fig. 8.**
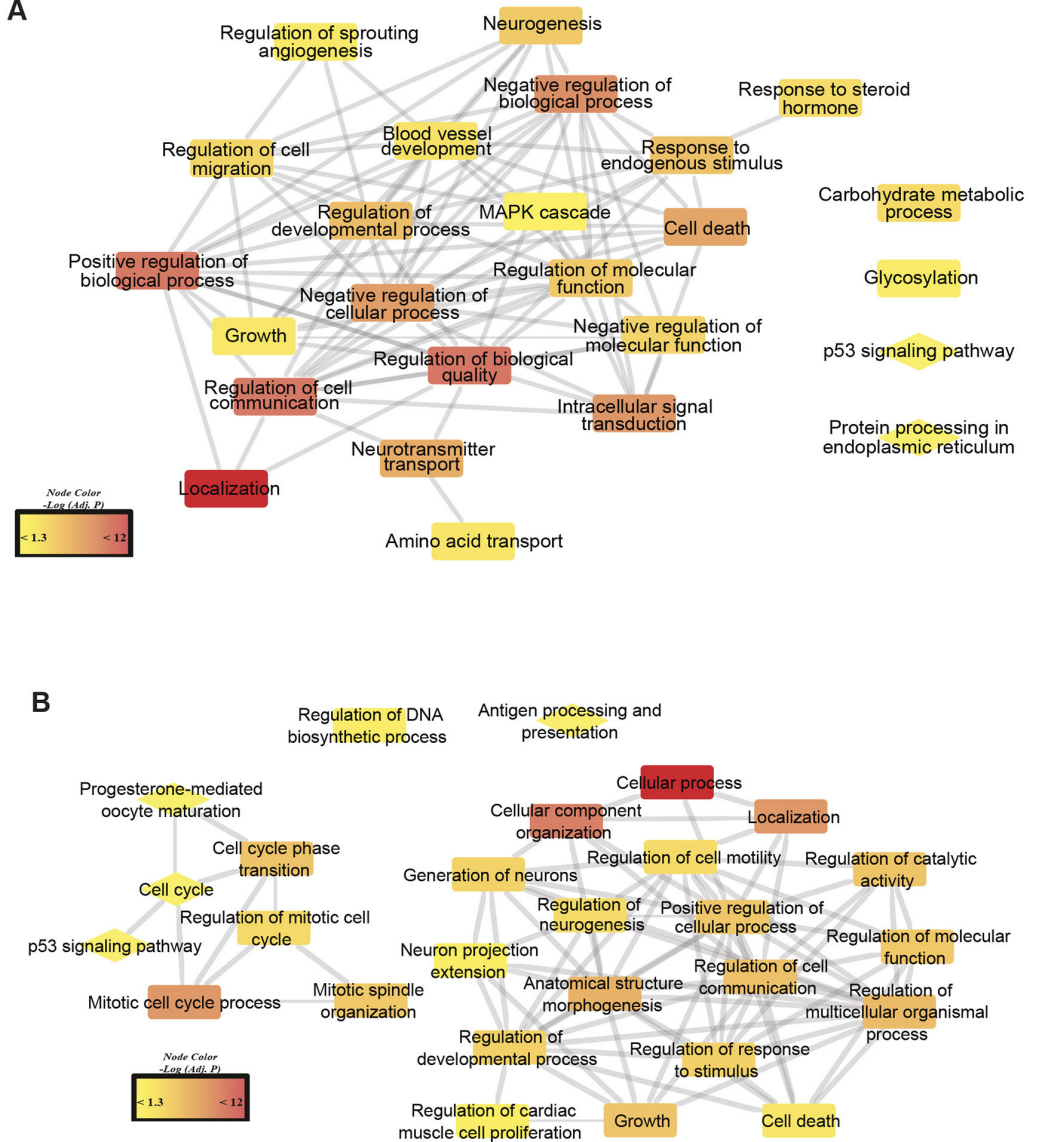
**Network of enriched pathways among shared DEGs across complication-prone tissue.** (A,B) Network analysis of differentially expressed genes (DEGs) shared between glomeruli and sciatic nerve in *db/+* STZ (streptozotocin; T1D) (A) and *db/db* (T2D) (B) mouse models were used to investigate overlapping functions across distinct tissues. Each network edge represents significant gene–content overlap between two nodes. Each node is overlaid with a symbol, with rectangles indicating Gene Ontology (GO) terms and diamonds indicating Kyoto Encyclopedia of Genes and Genomes (KEGG) pathways. The color range of the symbols (yellow to red) indicates increasing levels of significance (−log10-adjusted *P*-values).

To identify conserved changes between complication-prone tissues within diabetes types, network analysis was used to visualize pathways enriched with DEGs shared between Glom and SCN in *db/+* STZ T1D mice and *db/db* T2D mice. For *db/+* STZ animals (Fig. 8A), enriched pathways identified for both Glom and SCN primarily pertained to cellular metabolism and growth, signaling, migration or localization, and biological regulation (Tables S46-S48). A shared DEG of interest across Glom and SCN in *db/+* STZ was the kinase *Sphk1*. Network analysis of enriched pathways in Glom and SCN of *db/db* mice (Fig. 8B) similarly identified pathways related to cellular growth and localization or organization. However, immune-related antigen processing and presentation pathways, and neuron growth or extension pathways were also identified (Tables S49-S51). A shared DEG of interest across Glom and SCN for *db/db* was the cytokine receptor *Ifnlr1*.

### Correlations between DEGs of interest and parameters for DKD and DPN phenotyping

Finally, to determine whether the observed changes in gene expression are functionally meaningful and to identify differences across diabetes types, we performed correlation analyses between DEGs found in the Glom and SCN with DKD and DPN phenotyping parameters, respectively (Figs S7-S10). DEGs included in the analysis were those that had been identified as genes of interest during GO and KEGG analysis. In Glom, the global pattern of positive and negative correlations was similar in both diabetes types (Figs S7, S8). For both *db/+* STZ and *db/db* animals, the DEG *Mmp9* was negatively correlated with all DKD phenotype parameters, while *Sphk1* and *Ifnlr1* were positively correlated with all DKD parameters except ACR. In the SCN, the global pattern of positive and negative correlations was also similar in both diabetes types, but with some distinct differences (Figs S9, S10). Of note, sural NCVs were positively correlated with IENFDs in *db/+* STZ mice but not *db/db* animals. Additionally, the DEG of interest *Sphk1* was negatively correlated with IENFD in *db/+* STZ mice but positively correlated with IENFD in *db/db* animals. Finally, the strength of correlations between DEGs and phenotype parameters was higher in *db/+* STZ than *db/db* mice. Overall, the similar global patterns of correlation between the two diabetes types suggest that common pathogenic mechanisms occur in DKD and DPN in T1D as well as T2D.

## DISCUSSION

DKD and DPN are common diabetic complications but their underlying mechanisms remain poorly understood. Prior analyses provide useful information regarding DKD and DPN pathogenesis (Eid et al., 2019; Eid and Feldman, 2021; O'Brien et al., 2014), but high degrees of discordance were present in shared SCN DEGs in T1D versus T2D mouse models (Hur et al., 2016). Genetic background influences DKD and DPN development and progression (Gurley et al., 2010; Hinder et al., 2017a; Sullivan et al., 2007), necessitating studies on the same background. To address this need, we compared DKD and DPN transcriptomic data from T1D and T2D murine models on the same C57BLKS/J-*db/+* genetic background. SOM and DEG analyses followed by pathway enrichment analysis of Glom and SCN found that immune and inflammation pathways likely contribute to disease pathogenesis. Interestingly, there was a high degree of concordance in DEGs shared in T1D and T2D. Overall, these data offer important insight into potential pathogenic DKD and DPN pathways, emphasizing the role of inflammation and genetic background, regardless of diabetes type.

To compare DKD and DPN across T1D and T2D mouse models of the same genetic background, we first optimized an STZ-induced T1D mouse model. STZ injection, either as SHD or MLD, induces robust metabolic, renal and neuropathic changes of varying severity depending on dose (Furman, 2021; O'Brien et al., 2014; Tesch and Allen, 2007). As anticipated, SHD-STZ produced a more robust metabolic and neuropathic phenotype and was used for subsequent studies. Body weight was higher in *db/db* T2D versus *db/+* STZ T1D mice, consistent with humans; although, increasingly, T1D patients also develop obesity (Corbin et al., 2018). T1D and T2D mice developed DKD and DPN to a similar extent, although *db/db* mice had a slightly more robust DKD phenotype versus that of *db/+* STZ animals.

As in our prior studies, SOM and DEG analyses identified changes in immune- and inflammation-related pathways in both Glom and SCN of *db/+* STZ and *db/db* mice (Eid et al., 2021; Hinder et al., 2018, 2017b). Others report higher chemokine levels in glomeruli of DKD and spinal cord of DPN animal models (Chang and Chen, 2020; Sanajou et al., 2018; Sierra-Mondragon et al., 2018; Zychowska et al., 2015) together with macrophage recruitment (Saika et al., 2019; Sun et al., 2019; You et al., 2013). Indeed, we identified chemokine ligand *Ccl12* as a significant gene of interest in DKD and DPN, as previously in SCN (Elzinga et al., 2019; Hinder et al., 2018). CCL12 recruits inflammatory cells and regulates macrophage differentiation, and levels act as biomarkers for tubular injury in DKD (Hojs et al., 2015; Satirapoj, 2018). CCL2 overexpression in dorsal root ganglion neurons increases macrophage accumulation and neuron spreading (Niemi et al., 2016). Another inflammatory gene of interest in SCN of *db/db* mice was the phospholipase A2 family member *Pla2g2d* (Hinder et al., 2018). Members of this family generate bioactive lipid metabolites, which induce inflammatory cytokine production and secretion (Hui, 2012). Overall, these results continue to support the crucial role of the immune system and inflammation in DKD and DPN.

We also identified pathways related to the extracellular matrix in Glom of *db/+* STZ and *db/db* mice and SCN of *db/db* mice and the metalloproteinase *Mmp9* as a gene of interest within these pathways. During the repair process following acute kidney injury, MMP9 is activated and renal repair is impaired by MMP9 inhibition (Kaneko et al., 2012). However, in STZ T1D mice, blocking *Mmp9* reduces albuminuria and extracellular matrix accumulation, and improves peripheral nerve function and myelin sheath structure (Li et al., 2014; Ling et al., 2018; Wang et al., 2013; Yuan et al., 2022). Moreover, MMPs contribute to cytokine-mediated activation of proinflammatory pathways (Young et al., 2019), constituting another link to the immune system and inflammation. Overall, findings suggest that MMP9 is important to DKD and DPN pathogenesis, potentially through inflammation, and that it is a clinically relevant target.

In Glom, we observed downregulated RAS pathways, with *Cyp4a14* and *Cyp1a1* unique to T1D and T2D, respectively. CYP4 contributes to hypertension, renal fibrosis and DKD in animal models and humans (Eid et al., 2013; Ward et al., 2005; Zhou et al., 2018). CYP1A1 metabolizes estradiol and arachidonic acid to vasoactive compounds. Elevated CYP1A1 activity enhances hypertension risk (Lanca et al., 2005, 2002) and *Cyp1a1* gene polymorphisms have been linked to chronic kidney disease (Cheung et al., 1999; Siddarth et al., 2013). Downregulated RAS pathways in DKD may seem counterintuitive since hypertension is common in diabetic patients (Petrie et al., 2018) and increases DKD risk (Stamler et al., 1993). However, RAS dysregulation might be involved in inflammatory DKD progression through blood pressure-independent mechanisms, e.g. linked to increased cytokine production and end-organ damage (Feng et al., 2020; Lv and Liu, 2015). Thus, further investigation into the role of RAS in DKD pathogenesis, especially in the context of inflammatory responses, is warranted.

We previously reported that hyperglycemia might be a primary DKD driver (Hinder et al., 2017b; Hur et al., 2016), regardless of diabetes type (Hur et al., 2016). Here, significant changes in glycolysis, gluconeogenesis and insulin signaling were observed in Glom, and *Akt2* was a significant DEG in both *db/+* STZ and *db/db* mice. AKT2, a kinase with important roles in glucose and insulin metabolism, is dysregulated under insulin-resistant conditions. AKT2 signaling is renoprotective in T1D by enhancing antioxidant signaling with AMPK activation (Cheng et al., 2020) and promotes podocyte viability in chronic kidney disease (Canaud et al., 2013). These data suggest that *Akt2* is a therapeutic target in both T1D and T2D.

A goal of the current study was to pinpoint conserved pathways between complication-prone tissues to identify common therapeutic targets. In Glom and SCN, *Sphk1* was a DEG of interest in *db/+* STZ mice, whereas *Ifnlr1* was identified in *db/db* animals. The kinase SPHK1 regulates lipid metabolism and inflammation (Sukocheva et al., 2020; Wang et al., 2019), both of which play prominent roles in DPN pathogenesis (Afshinnia et al., 2022; Guo et al., 2022; Hinder et al., 2018; Hur et al., 2016; O'Brien et al., 2020; Rumora et al., 2021, 2022). This suggests that lipid metabolism and inflammation also participates in DKD in T1D. Indeed, *Sphk1* correlated positively with all DKD parameters except ACR. Little is known regarding the role of IFNLR1, a cytokine receptor that binds interleukins, in DKD and DPN. However, it recently received attention as a potential T2D biomarker (Gummesson et al., 2021) and predictive marker for response to metformin treatment (Zhong et al., 2021). Additionally, single nucleotide polymorphisms in the IFNLR1 signaling pathway are linked to risk of chronic kidney disease (Kwak et al., 2022). Here, *Ifnlr1* correlated positively with all DKD parameters, except ACR, and negatively with SCN NCVs in *db/db* mice, indicating that IFNLR1 pathways drive DKD and DPN in T2D. Involvement of both *Sphk1* and *Ifnlr1* again suggest immune system dysregulation in both DKD and DPN pathogenesis in T1D and T2D.

Studies support a role for genetics in DKD (Bufi and Korstanje, 2022; Gu, 2019; Gurley et al., 2018, 2010). DPN studies have similarly identified candidate genes influencing pathogenesis, implicating genetic factors (Babizhayev et al., 2015; Bedlack et al., 2003; Ezzidi et al., 2008; Yigit et al., 2013). However, results are inconsistent across studies and fail to explain underlying DPN mechanisms (Jankovic et al., 2021; Witzel et al., 2015). Previously, we found a high degree of concordance in DEGs shared between T1D and T2D mouse models on different genetic backgrounds in Glom (94%) but not in SCN (54%) (Hur et al., 2016). In this current study, both T1D and T2D mouse models were on a C57BLKS/J-*db/+* background, which moderately enhanced concordance in Glom (99.6%) and markedly enhanced concordance in SCN (97.7%). This is of particular interest for DPN, for which a role for genetic factors remains incompletely characterized.

We found DKD phenotype parameters to be correlated with DEGs in a similar pattern in either diabetes type, supporting conserved pathologic mechanisms between T1D and T2D (Hodgin et al., 2013; Hur et al., 2016). Our previous work has suggested differing mechanisms underlying DPN pathogenesis by diabetes type (Hur et al., 2016). In this study, a few DPN parameters differed in our correlation analysis by diabetes type. First, sural NCVs correlated positively with IENFDs in T1D but not T2D. This suggests a similar extent of small and large fiber dysfunction and more global nerve damage in T1D versus T2D. Second, expression of *Sphk1* correlated positively with IENFDs in T2D but negatively in T1D, highlighting that pathways underlying DPN pathogenesis in T1D and T2D are not completely identical. However, like DKD, overall global correlation patterns were similar for both types of diabetes, suggesting that genetic background influences the pathogenesis of these complications. Future studies should focus on elucidating the role of genetic background, particularly by using mouse models that better reflect the heterogeneity of human genetics to improve translation significance.

The current study used bulk RNA sequencing, which aggregates transcriptomics from multiple cell types, but future mechanistic studies would benefit from cell-specific insight. Additionally, novel data from *db/+* STZ mice were compared with historic phenotyping and transcriptomic datasets from *db/+* and *db/db* animals. However, although similarities across datasets support their comparison, they were not studied contemporaneously. Finally, a larger sample size may identify additional statistically significant relationships, particularly when there are multiple comparisons across diabetes and strain type. In summary, this study provides insight into differences and similarities of complication-prone tissues, head-to-head in T1D and T2D models. We detected dysregulated DEGs in pathways related to immunity in both T1D and T2D, confirming our previous findings and pinpointing potential therapeutic targets. Glom and SCN DEG datasets were highly concordant in T1D and T2D mouse models of the same genetic background, suggesting that genetic background influences diabetic complications, which is novel regarding DPN.

## MATERIALS AND METHODS

### Animals

Data were obtained from two cohorts of mice.

Cohort 1 was used to identify the optimal STZ paradigm to induce a robust T1D DPN phenotype. Cohort 1 comprised 4-week-old male C57BLKS/J-*db/+* mice (BKS.Cg-*Dock7^m^* +/+ *Lepr^db^*/J; strain #000642; The Jackson Laboratory, Bar Harbor, ME, USA). STZ (Millipore Sigma, St. Louis, MO, USA; product no. S0130) was prepared in citrate buffer (pH 4.5), and 5-week-old mice were randomly administered a single high dose (SHD) of STZ (150 mg kg^−1^ STZ; SHD-STZ), multiple low doses (MLD) of STZ (individual doses of 50 mg kg^−1^ for five consecutive days; MLD-STZ) or a control dose (citrate buffer; *db/+* Ctrl) as an intraperitoneal injection (*n*=8 mice/group) (Clodfelder-Miller et al., 2006; Planel et al., 2007). MLD of STZ were given according to the established Animal Models of Diabetic Complications Consortium (AMDCC) protocol as a daily intraperitoneal injection for five consecutive days (Sullivan et al., 2007). Animals with a glycemic level >300 mg dL^−1^ were considered diabetic. Mice were killed at 16 weeks of age following phenotyping. Based on the phenotype most closely mimicking humans, the SHD-STZ mouse model was chosen as the T1D paradigm moving forward.

Cohort 2 was used for metabolic and tissue-specific phenotyping of the *db/+* T1D mouse model and for transcriptomic analysis of Glom and SCN tissues. Cohort 2 consisted of 4-week-old male C57BLKS-*db/+* (BKS.Cg-m+/+Lepr^db^/J; strain #000642; The Jackson Laboratory) mice. *db/+* Ctrl mice were given a SHD of STZ (150 mg kg^−1^ STZ) at 6 weeks of age to establish a T1D model (*db/+* STZ, T1D, *n*=6). Terminal phenotyping and sample collection were performed at 16 weeks of age as outlined in the phenotyping method subsections.

All mice were housed in a pathogen-free environment and kept at a temperature of 20±2°C with a 12:12 h light:dark cycle. Water and food were available *ad libitum*, and mice were monitored by the University of Michigan Unit for Laboratory Animal Medicine. Animals were assigned a unique ID and investigators were blinded to experimental conditions when possible. Mice were killed as previously published (Kim et al., 2011). All procedures complied with Diabetic Complications Consortium protocols and were approved by the University of Michigan University Committee on Use and Care of Animals.

This study also analyzed data of *db/+* Ctrl (*n*=6) and *db/db* (T2D, *n*=6) mice that had undergone metabolic, DKD and DPN phenotyping, and transcriptomic analyses in a previous study (Hinder et al., 2017b).

### Metabolic phenotyping

Diabetic phenotype was established with weekly body weights, and by measuring fasting blood glucose levels (4 h) and plasma metabolite concentrations. Plasma analytes were measured at multiple time points depending on the experiment and are detailed in Figs S1 and S4; they included plasma insulin, glycated hemoglobin (GHb), total cholesterol and total triglycerides. Fasting blood glucose levels were measured using an AlphaTrak glucometer (Abbott Laboratories, Abbott Park, IL, USA). Readings above the 750 mg dL^−1^ range of the glucometer were arbitrarily set at 750 mg dL^−1^ for statistical analysis. Plasma insulin, total triglyceride and total cholesterol concentrations were measured by the Mouse Metabolic Phenotyping Center (MMPC; Vanderbilt University, Nashville, TN, USA; University of Cincinnati, Cincinnati, OH, USA). Blood GHb concentrations were measured using a Glyco-Tek Affinity column (Helena Laboratories, Beaumont, TX, USA) by the Michigan Diabetes Research Center's Chemistry Core.

### DKD phenotyping

Animals were phenotyped for diabetic kidney disease (DKD) according to the guidelines by the Diabetic Complications Consortium, for urinary albumin-to-creatinine ratio (ACR) and kidney histopathology (glomerular hypertrophy and mesangial index), as previously published (Hinder et al., 2017b). Kidney histopathology was scored in left kidney tissue isolated from mice following phosphate buffered saline (PBS) perfusion at study termination. Briefly, after systemic PBS perfusion, the left kidney was excised, fixed, sectioned, stained using the periodic acid-Schiff (PAS) method and imaged using a digital camera. Glomerular tufts for analysis were randomly chosen per animal. Mesangial area was quantified with MetaMorph (v.7.7.0.8, Molecular Devices) by determining the percentage of the total glomerular area that was stained using the PAS method. ACR was assessed by measuring urinary albumin and creatine concentrations collected from mice on day 3 following a 3-day stay in metabolic cages, using Albuwell M and Creatinine Companion assays, respectively (Exocell, Philadelphia, PA, USA).

### DPN phenotyping

Animals were phenotyped for diabetic peripheral neuropathy (DPN) according to the guidelines by the Diabetic Complications Consortium (Biessels and Kappelle, 2005; Biessels et al., 2002). Previously published protocols were used to determine hind paw withdrawal latencies as a measure of thermal sensitivity (Lee et al., 1990; Sullivan et al., 2007). Briefly, the temperature under a red-light emitter below the hind paw was incrementally increased from 25°C to 55°C over the course of 20 s. Total time between activation of the beam and hind paw withdrawal was recorded to measure withdrawal latency (Lee et al., 1990). Six measurements per mouse were carried out and an average latency was reported.

Large nerve fiber function was determined by measuring nerve conduction velocities (NCVs) and through electrophysiological testing of sural sensory and sciatic-tibial motor NCVs (Oh et al., 2010; Stevens et al., 1994). Briefly, measurements were taken using stainless steel needle electrodes (Natus Medical, San Carlos, CA, USA) from animals that were kept anesthetized under isoflurane with body temperature held at 34°C with a heating lamp. Sural sensory NCVs were recorded at the dorsum of the foot following antidromic supramaximal stimulation at the ankle. The NCV (m/s) was calculated by dividing the distance between the ankle and the foot by the onset latency of the sensory nerve action potential. Similarly, sciatic-tibial motor NCVs were recorded at the dorsum of the foot after orthodromic supramaximal stimulation at the ankle and then at the sciatic notch. Latencies were measured for each (ankle and sciatic notch) at the initial onset of compound muscle action potential. To calculate the sciatic-tibial motor NCVs, the measured ankle distance was subtracted from the measured notch distance and divided by the difference between ankle and notch onset latencies.

Intraepidermal nerve fiber density (IENFD) was determined using published protocols (Cheng et al., 2012; Sullivan et al., 2007). In brief, plantar surface hind-paw footpads were removed, fixed, washed, embedded and cryo-preserved until sectioning. For immunohistochemistry, 30-μm sections were labeled with PGP9.5 (Cat. no. 14730-1-ap, Proteintech, Rosemont, IL, USA). Three *z*-series images were taken using a confocal microscope (Leica SP5, 20×1.2 water-immersion objective, 1024×1024 pixel resolution; Leica Microsystems, Wetzlar, Germany). MetaMorph (v.7.7.0.8, Molecular Devices) software was used to determine the number of fibers per mm^2^ of epidermis.

### RNA-sequencing analysis

Total RNA was isolated from Glom and SCN at 16 weeks of age from *db/+* SHD-STZ mice in Cohort 2 using a RNeasy Mini Kit (Qiagen, Germantown, MD, USA). RNA quality was evaluated using TapeStation (Agilent, Santa Clara, CA, USA) and samples with an RNA-integrity number of ≥8 were processed with a TruSeq mRNA Sample Prep v2 kit (cat. nos: RS122-2001, RS-122-2002; Illumina, San Diego, CA, USA). Single-end 100 bp reads were obtained using an Illumina HiSeq-2000 sequencer by the University of Michigan DNA Sequencing Core. Raw sequencing read files for *db/+* Ctrl and *db/db* mice from both Glom and SCN were obtained from our previously published study [Hinder et al., 2017b; Gene Expression Omnibus (GEO) accession ID: GSE123853] and analyzed again, together with the current *db/+* STZ dataset.

Quality control of the RNA-sequencing data was assessed using the Babraham Bioinformatics FastQC tool. Raw sequencing reads were cleaned using Trimmomatic (Bolger et al., 2014) to remove reads with adapter or Poly-N sequences or a quality score of <30. Cleaned reads were aligned to the mouse Genome Reference Consortium Mouse Build 38 patch release 6 (GRCm38.p6) using HISAT2 (Kim et al., 2015). featureCounts (Liao et al., 2014) was used to count uniquely mapped reads, and genes with zero expression across samples were omitted from differential expression analysis. Fragments per kilobase of exon per million mapped reads as a measurement of transcript expression were calculated using the fpkm() function in the DESeq2 R package.

### SOM analysis

SOM analysis was used to identify gene expression patterns in an unbiased manner across diabetes type in Glom and SCNs (Eid et al., 2021; Hinder et al., 2018). To compare gene expression changes across groups, count values were normalized by subtracting the mean count of all samples from the mean count value of each group. Normalized gene counts were sorted into modules with similar expression patterns within a 5×5 grid structure using the Kohonen R package (Wehrens and Buydens, 2007; Wehrens and Kruisselbrink, 2018). Modules were further grouped through hierarchical clustering to identify closely related modules and modules with the most robust expression differences between groups. Module clusters were analyzed for functional enrichment using the in-house R package, richR, Gene Ontology (GO) (Mi et al., 2017) and Kyoto Encyclopedia of Genes and Genomes (KEGG) (Kanehisa et al., 2017, 2019; Kanehisa and Goto, 2000). GO terms and KEGG pathways with a Benjamini–Hochberg-corrected *P*-values (*P*<0.05) were deemed to be significantly enriched. GO terms were further clustered using Kappa statistics (Huang et al., 2009a,b) to identify non-redundant as well as representative and significantly enriched canonical pathways.

### DEG analysis and pathway enrichment

DEGs between *db/+* STZ versus *db/+* Ctrl and *db/db* versus *db/+* Ctrl groups for both Glom and SCN were identified using the DESeq2 R package (Love et al., 2014). All genes with an adjusted *P*<0.05 were deemed to be differentially expressed. These DEG lists were used as input in richR to identify enriched biological functions and pathways. Genes were determined to be unique or shared by using our in-house gene-set overlap analysis R package VennDetail, and separated into their respective groups based on tissue and animal model as illustrated in Fig. 1. Lists of genes both unique and shared between datasets were also assessed for enrichment of functional terms and pathways.

### Network analysis

Enrichment results using two different annotations sets were combined and visualized in a network by richR, where edges indicate significant overlap in terms of gene content belonging to two nodes (biological function and pathways). Enriched GO terms were filtered to remove redundancy as described above and the top significant terms based on Benjamini–Hochberg-adjusted *P*-values from each cluster were used. These top GO cluster terms were used together with significantly enriched terms based on adjusted *P*-value from KEGG, yielding the nodes of the generated networks for each dataset. The edges of the network were composed of the shared genes between terms to demonstrate the connections between nodes. Cytoscape (Shannon et al., 2003) was used to visualize the network and colorize nodes based on their *P*-value. All networks were organized using the organic yFiles layout in Cytoscape with minimal manual node rearrangement for improved visibility while maintaining node grouping.

### Previous datasets

Metabolic, DKD and DPN (except IENFD) phenotyping, and Glom and SCN transcriptomic datasets at 16 weeks of age were collected from *db/+* Ctrl and *db/db* mice in our previous study (Hinder et al., 2017b). Data corresponding to *db/+* Ctrl and *db/db* mice were used in this current study with the newly presented *db/+* STZ phenotyping and transcriptome data for comparative analyses.

### Statistical analyses

The proposed mouse group sizes were calculated to achieve 90% power and 95% sensitivity to identify statistically significant differences between groups in the ACRs for DKD and in IENFDs and NCVs for DPN (Hinder et al., 2017a; O'Brien et al., 2020, 2018). GraphPad Prism v.7 (GraphPad Software, La Jolla, CA, USA) was used to complete statistical analyses. When applicable, Tukey's or Bonferroni's post-hoc test for multiple comparisons (Festing and Altman, 2002) were applied following one-way ANOVA for cross-sectional comparisons. The data underwent a base-2 logarithmic transformation when not normally distributed as determined by a Brown–Forsythe test. If data were not normal even after log2 transformation, non-parametric tests were applied in the form of Kruskal–Wallis testing. Multiple regression and correlation analyses were conducted using R Statistical Software. Multiple regression analyses were performed sequentially by first examining the differences between treatments (*db/+* Ctrl, *db/+* STZ, and *db/db*) and then assessing the correlation of DKD and DPN phenotyping with related DEGs of interest. Significance was set at nominal *P<*0.05 and results represented as least square mean±s.e.m. for normally distributed data, or median with interquartile range for non-normally distributed data.

## Supplementary Material

10.1242/dmm.050080_sup1 Supplementary information
