# Supplementary material for: Transcriptomic analysis of diabetic kidney disease and neuropathy in mouse models of type 1 and type 2 diabetes
Source: Dis Model Mech. 2023 Oct 4;16(10):dmm050080. doi: 10.1242/dmm.050080 (PMC10565109; doi:10.1242/dmm.050080)
Supplement: Supplementary information [file dmm-16-050080-s1.pdf]

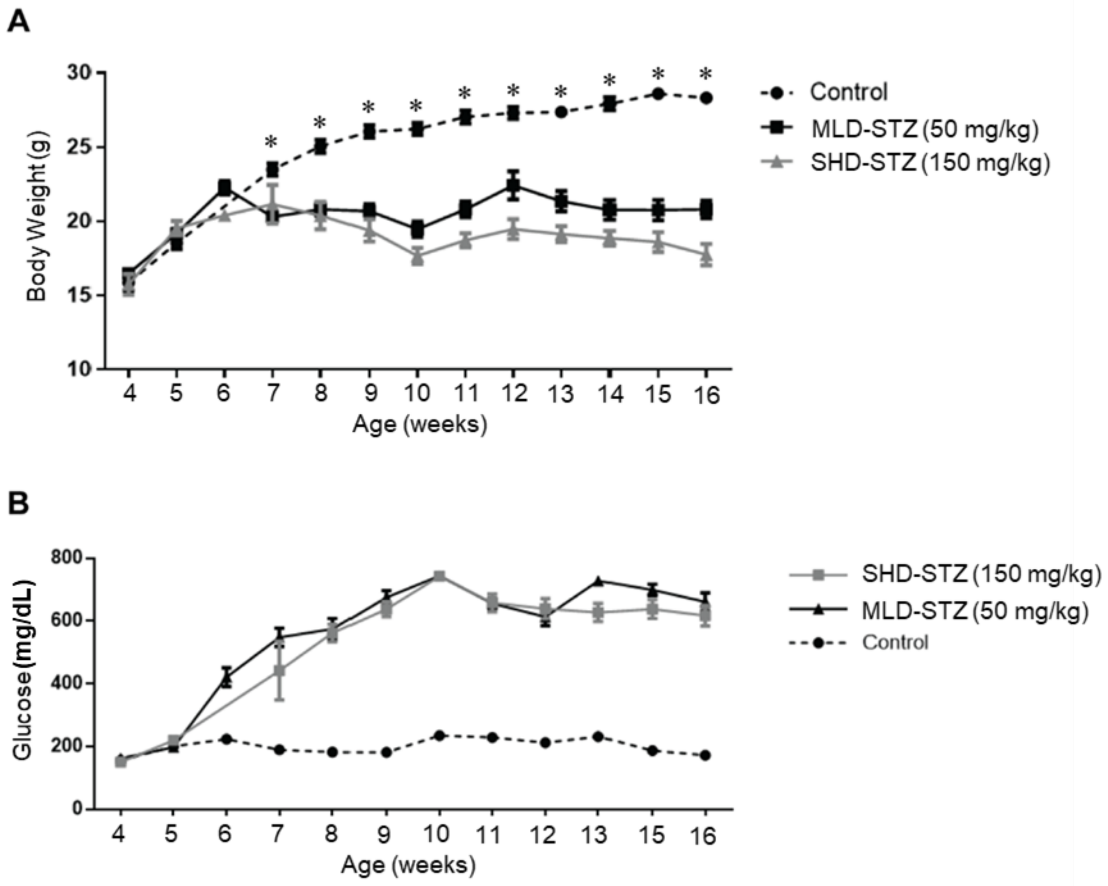

Fig. S1. Metabolic phenotyping of STZ-induced mice. Weekly body weights (**A**) and plasma glucose concentrations (**B**) in controls (*db/*+ Ctrl, n = 8), mice given MLD-STZ (*db/*+ STZ (50 mg kg^{-1}), n = 8), and mice given SHD-STZ (*db/*+ STZ (150 mg kg^{-1}), n = 8). Data are represented as least square means ± standard error of the mean. From week 7 forward, control mice weighed significantly more (*$p < 0.05$) compared to SHD-STZ and MLD-STZ animals.

Disease Models & Mechanisms • Supplementary information

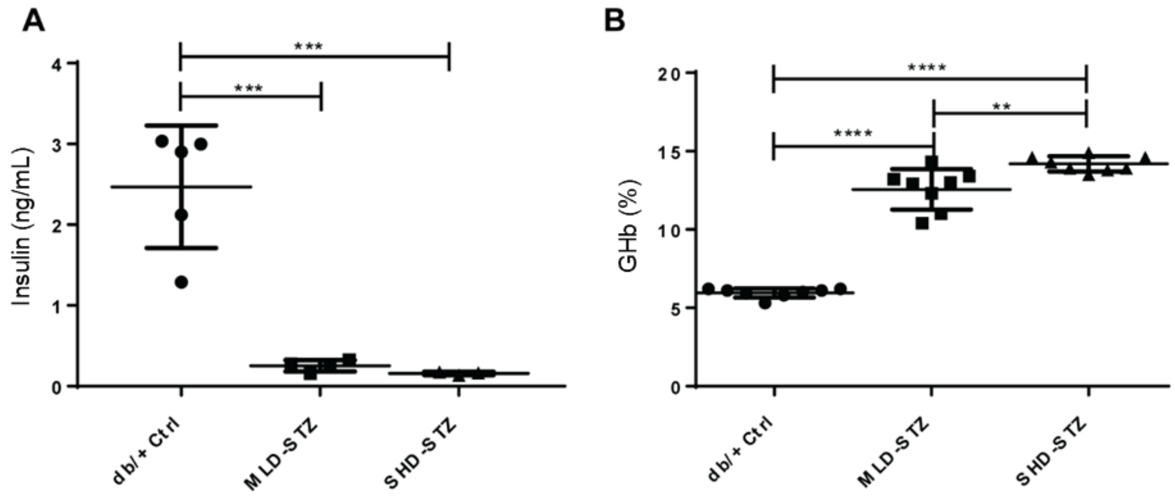

Fig. S2. Metabolic phenotyping of STZ-induced mice. Plasma insulin (**A**) and glycated hemoglobin (GHb) (**B**) concentrations after 16 weeks in control mice (*db/+* Ctrl, n = 5-8), mice administered MLD-STZ (*db/+* STZ (50 mg kg^{-1}), n = 4-8), and mice administered SHD-STZ (*db/+* STZ (150 mg kg^{-1}), n = 3-8). Data are represented as least square mean ± the standard error of the mean. Statistically significant $^{**}p < 0.01$, $^{***}p < 0.001$, $^{****}p < 0.0001$.

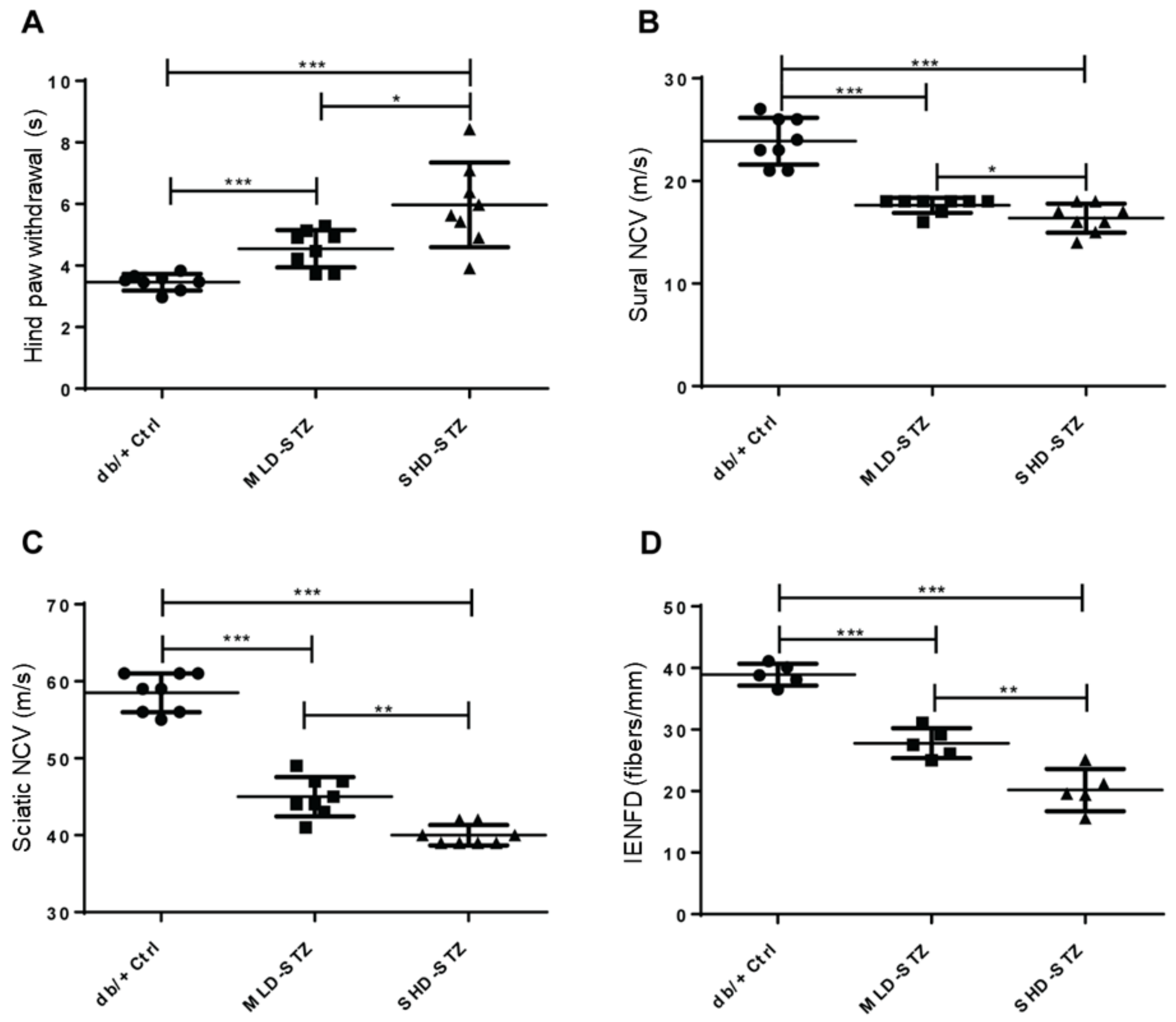

Fig. S3. DPN phenotyping of STZ-induced mice. Measures of neuropathy as determined by hind paw withdrawal (**A**), sural nerve conduction velocity (NCV) (**B**), sciatic NCV (**C**), and intraepidermal nerve fiber density (IENFD) (**D**) at 16 weeks in control mice (*db/*+ Ctrl, n = 5-8), mice administered MLD-STZ (*db/*+ STZ (50 mg kg^{-1}), n = 5-8), and mice administered SHD-STZ (*db/*+ STZ (150 mg kg^{-1}), n = 5-8). Data are represented as least square mean ± the standard error of the mean. Statistically significant $*p < 0.05$, $**p < 0.01$, $***p < 0.001$.

Disease Models & Mechanisms • Supplementary information

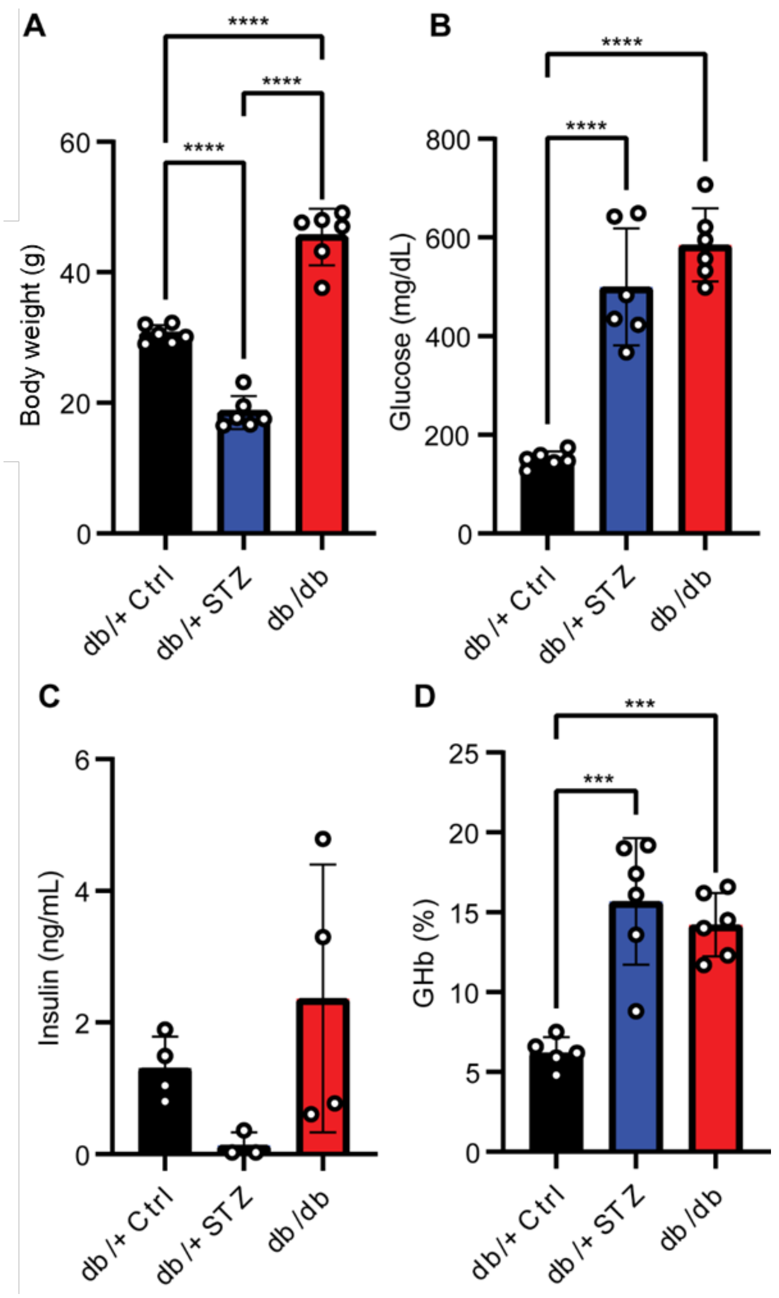

Fig. S4. Metabolic phenotyping of T1D and T2D mouse models. Measures of metabolic impairment as determined by body weight (A), fasting blood glucose (B), insulin (C), and glycated hemoglobin (GHb) (D) in *db/*+ Ctrl, *db/*+ STZ (T1D) and *db/db* (T2D) mice (n = 3-6/group) at 16 weeks. Data are represented as least square mean ± the standard error of the mean. Statistically significant ***$p < 0.001$, ****$p < 0.0001$. Data from *db/*+ Ctrl and *db/db* mice were previously published in Hinder *et al.*, 2017.

Disease Models & Mechanisms • Supplementary information

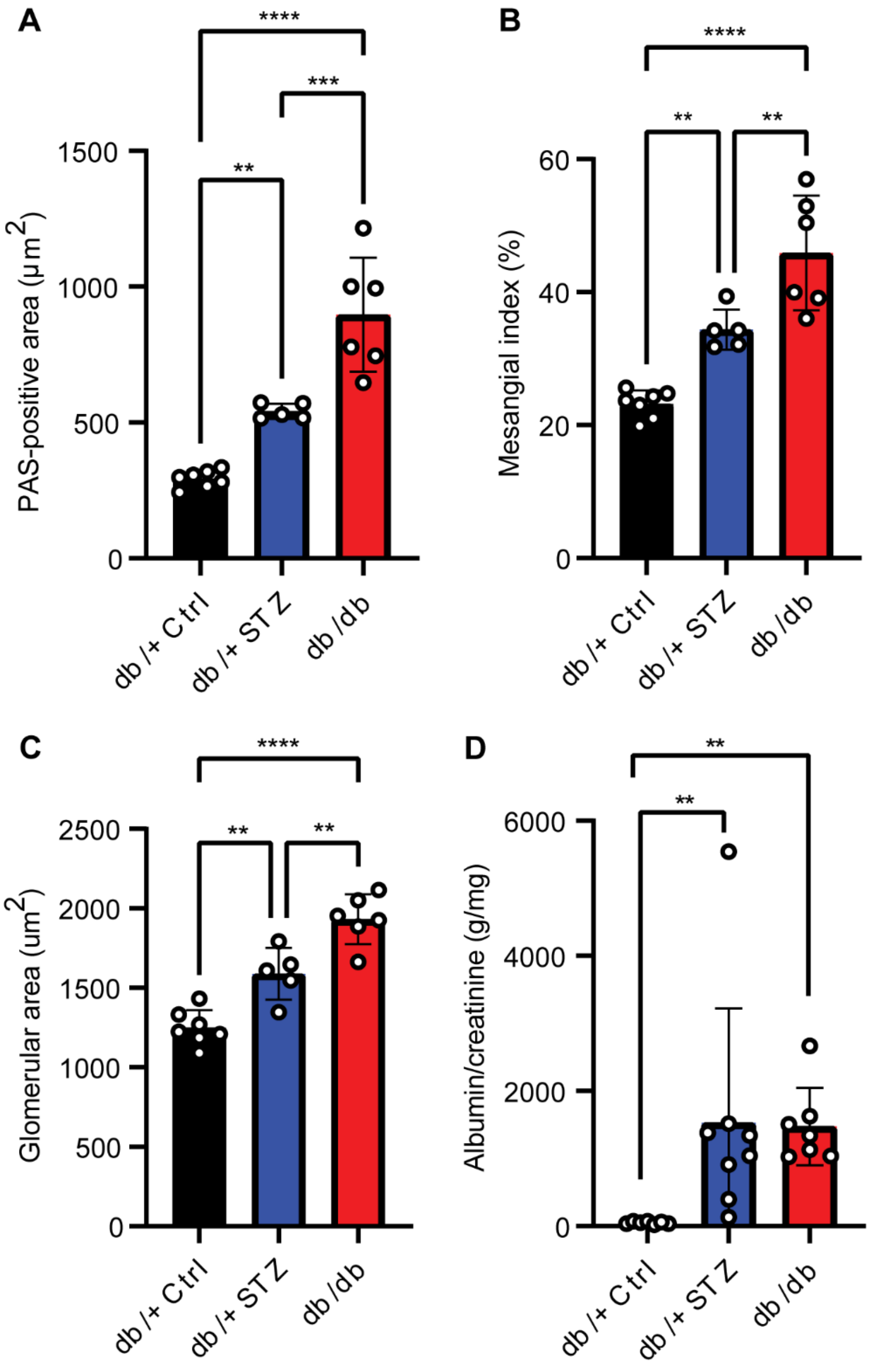

Fig. S5. DKD phenotyping of T1D and T2D mouse models. Measures of nephropathy as determined by periodic acid-Schiff (PAS)-positive area (A), mesangial index (B), glomerular area (C), and albumin/creatinine ratio (D) in *db/*+ Ctrl, *db/*+ STZ (T1D) and *db/db* (T2D) mice (n = 4-6/group) at 16 weeks. Data are represented as least square mean ± the standard error of the mean. Data are represented as least square mean ± the standard error of the mean. Statistically significant $^{**}p < 0.01$, $^{***}p < 0.001$, $^{****}p < 0.0001$. Data from *db/*+ Ctrl and *db/db* mice were previously published in Hinder *et al.*, 2017.

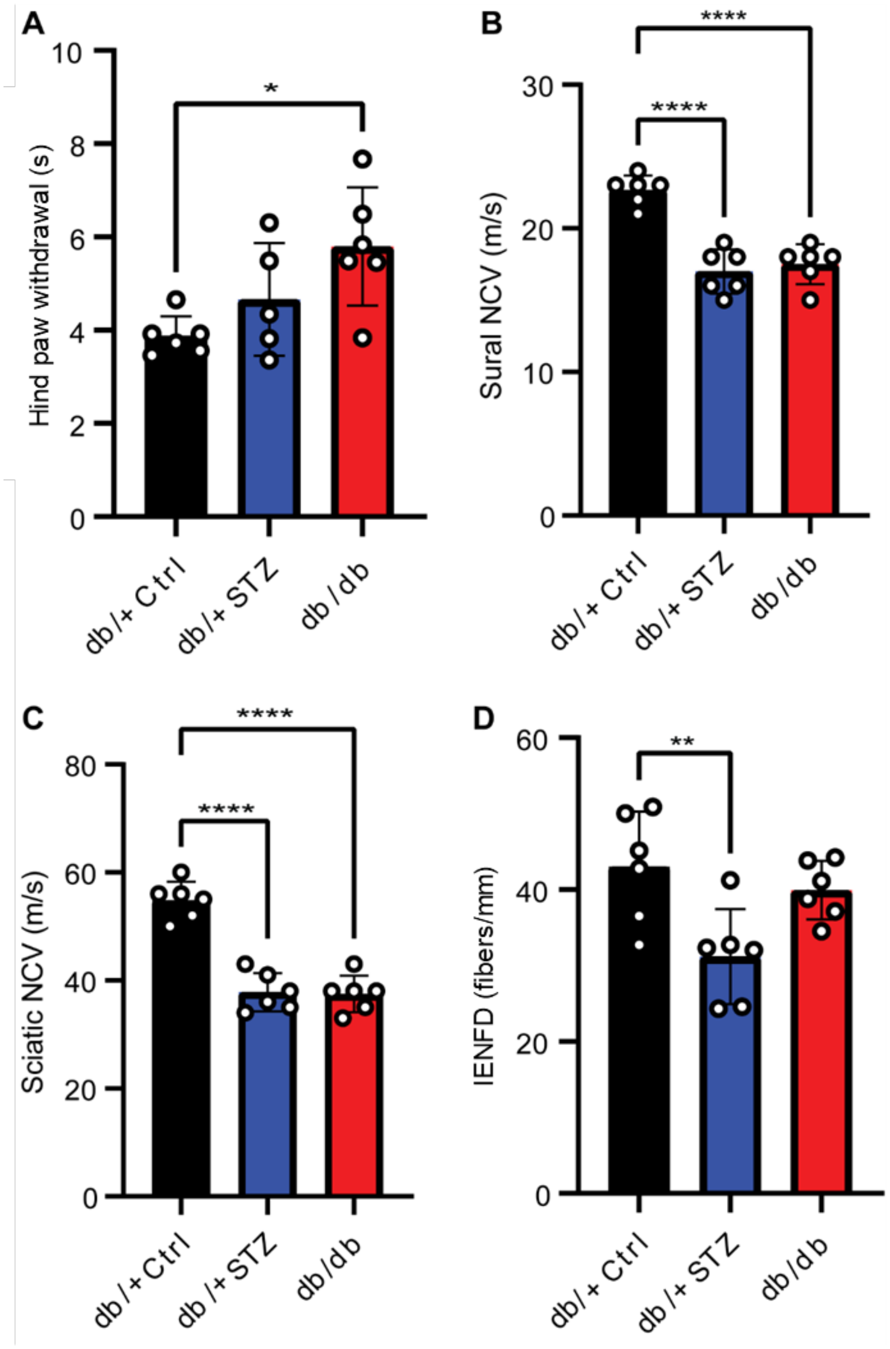

Fig. S6. DPN phenotyping of T1D and T2D mouse models Measures of neuropathy as determined by hind paw withdrawal latency (A), sural nerve conduction velocity (NCV) (B), sciatic NCV (C), and intraepidermal nerve fiber density (IENFD) (D) in *db/*+ Ctrl, *db/*+ STZ (T1D) and *db/db* (T2D) mice (n = 5-6/group) at 16 weeks. Data are represented as least square mean ± the standard error of the mean. Statistically significant $^{*}p < 0.05$, $^{**}p < 0.01$, $^{****}p < 0.0001$. Data from *db/*+ and *db/db* mice were previously published in Hinder *et al.*, 2017.

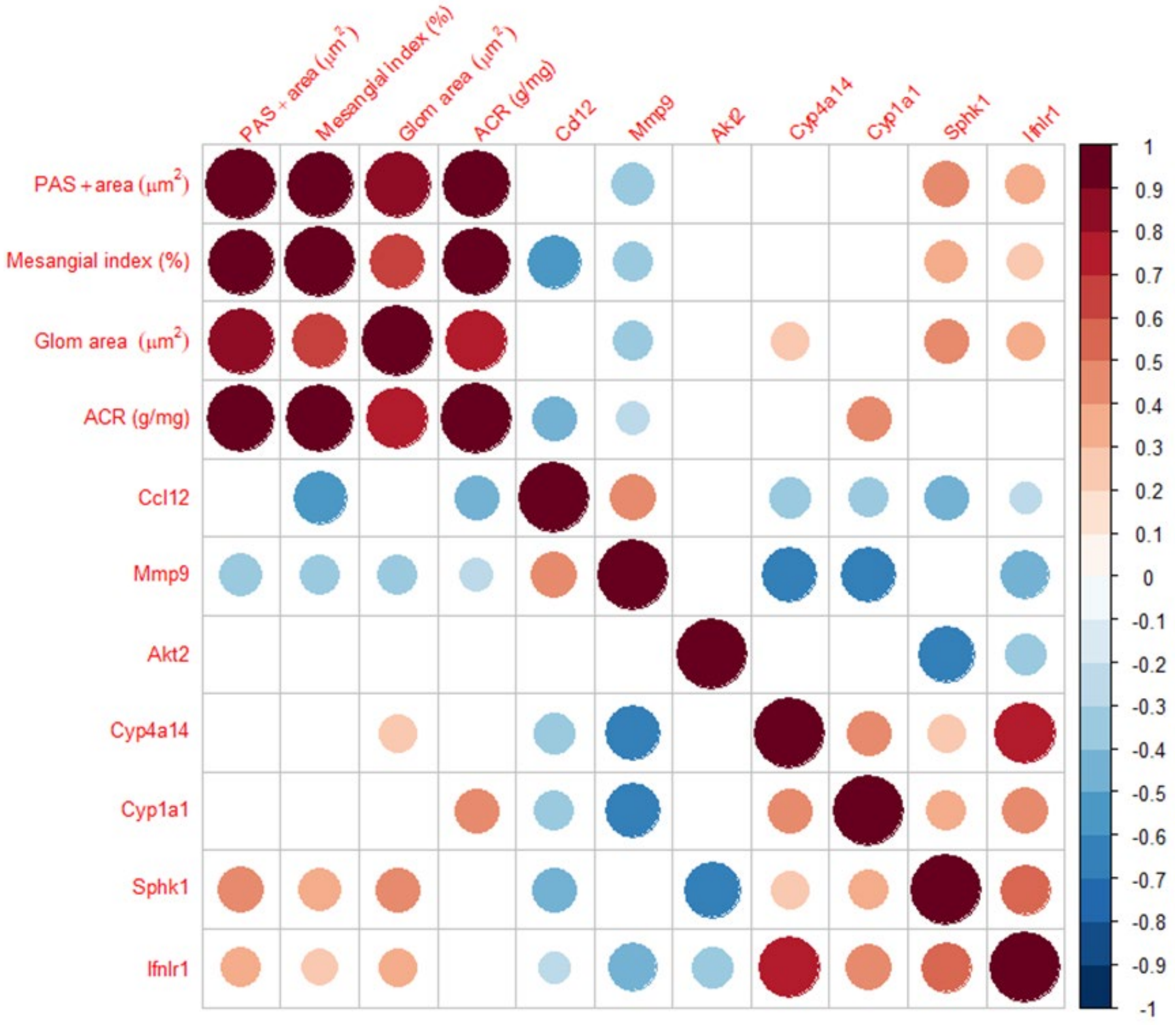

Fig. S7. Correlation between DKD parameters and DEGs of interest from T1D mice. Plot of pair-wise Pearson correlation of DKD parameters and differentially expressed genes of interest from the Glom of *db/*+ Ctrl and *db/*+ STZ T1D mice (n = 4-6/group). Positive correlations are represented in red and negative correlations in blue. Darkness of color and large circle size indicate a strong correlation. Factors are sorted by clusters and factors with strong positive correlations grouped to the same cluster. Significance was set at $p < 0.05$. Insignificant correlations are blank.

Disease Models & Mechanisms • Supplementary information

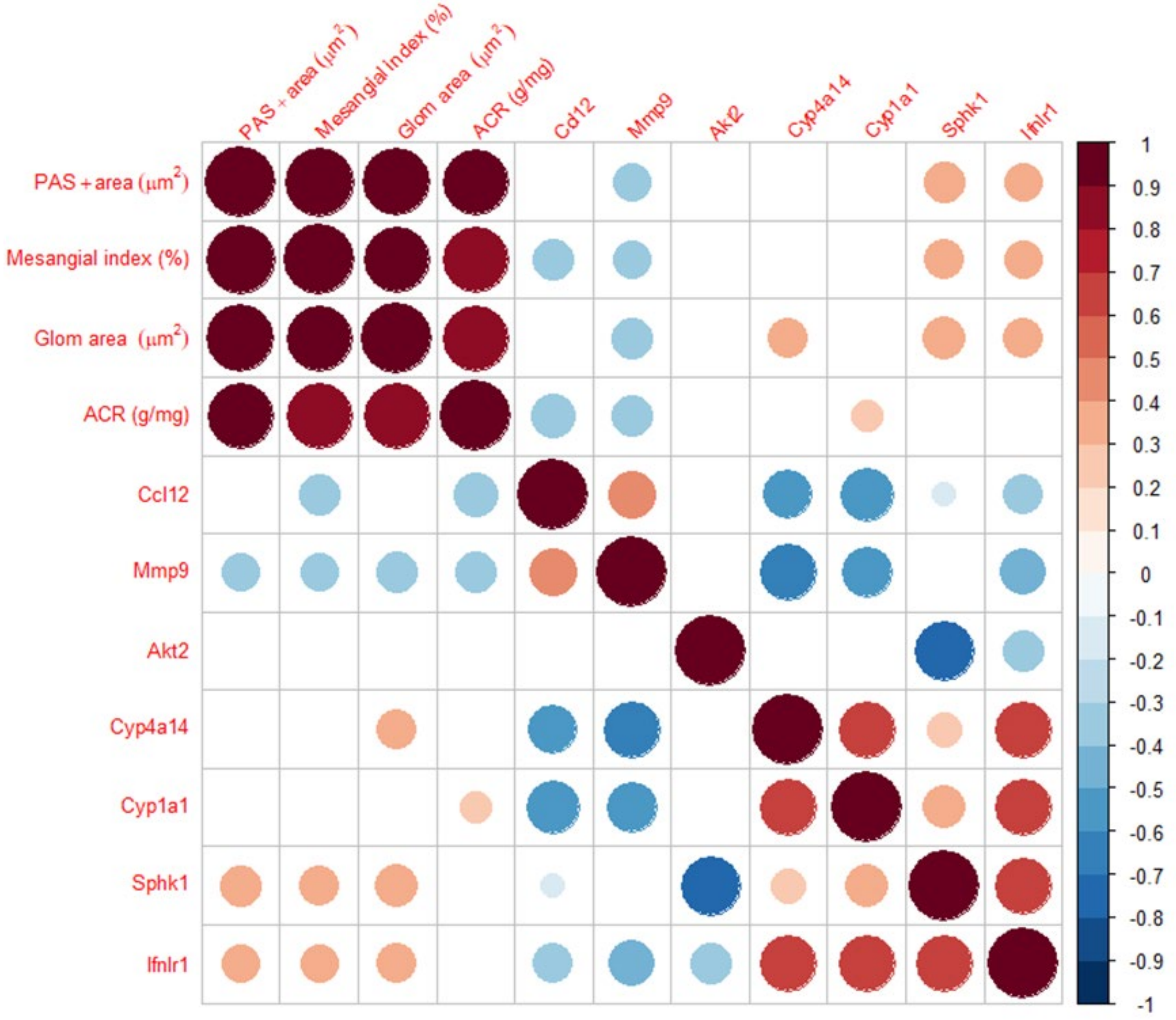

Fig. S8. Correlation between DKD parameters and DEGs of interest from T2D mice. Plot of pair-wise Pearson correlation of DKD parameters and differentially expressed genes of interest from the Glom of *db/+* Ctrl and *db/db* T2D mice (n = 4-6/group). Positive correlations are represented in red and negative correlations in blue. Darkness of color and large circle size indicate a strong correlation. Factors are sorted by clusters and factors with strong positive correlations grouped to the same cluster. Significance was set at $p < 0.05$. Insignificant correlations are blank.

Disease Models & Mechanisms • Supplementary information

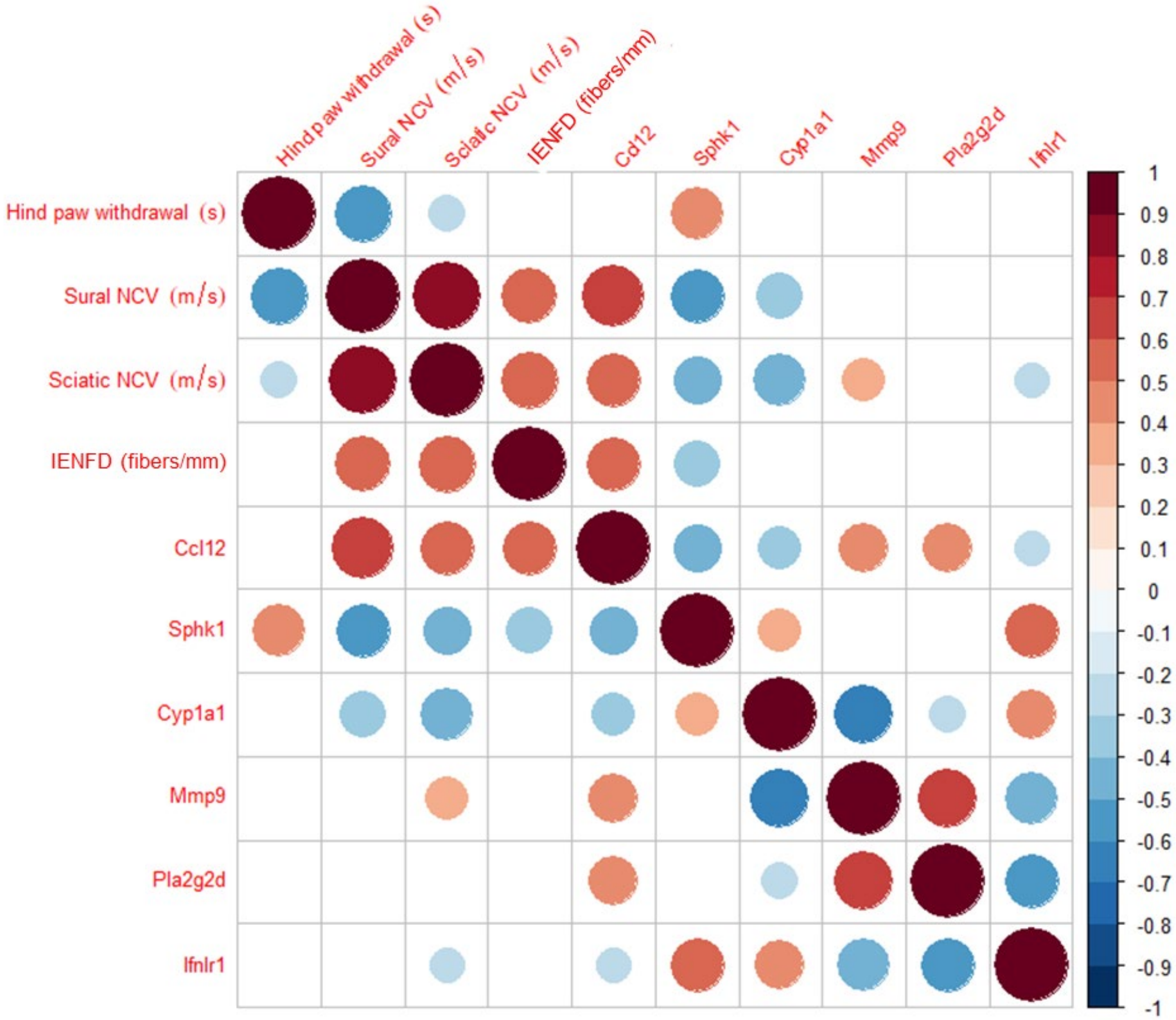

Fig. S9. Correlation between DPN parameters and DEGs of interest from T1D mice. Plot of pair-wise Pearson correlation of DPN parameters and differentially expressed genes of interest from the SCN of *db/+* Ctrl and *db/+* STZ T1D mice (n = 5-6/group). Positive correlations are represented in red and negative correlations in blue. Darkness of color and large circle size indicate a strong correlation. Factors are sorted by clusters and factors with strong positive correlations grouped to the same cluster. Significance was set at $p < 0.05$. Insignificant correlations are blank.

Disease Models & Mechanisms • Supplementary information

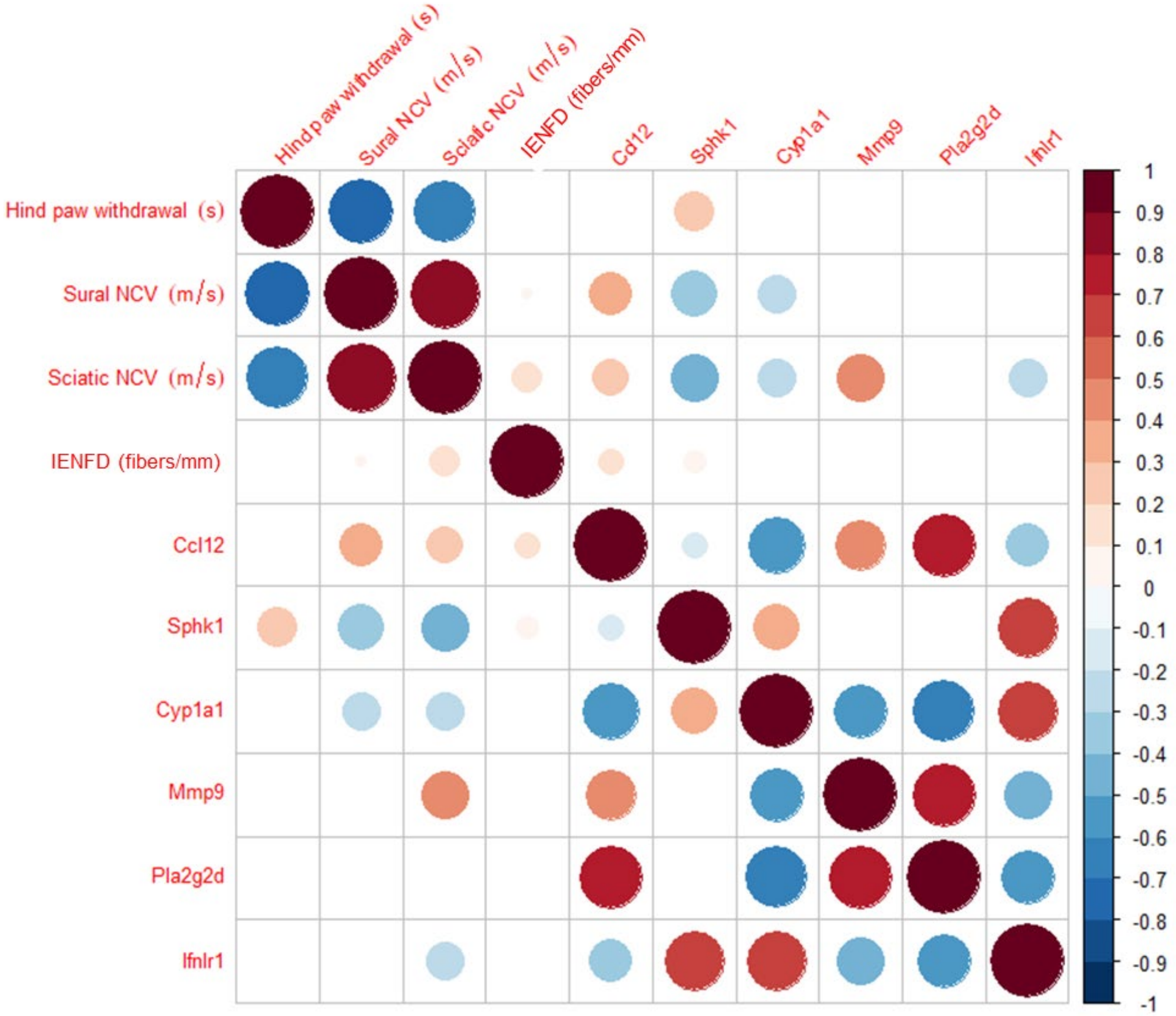

Fig. S10. Correlation between DPN parameters and DEGs of interest from T2D mice. Plot of pair-wise Pearson correlation of DPN parameters and differentially expressed genes of interest from the SCN of *db/+* Ctrl and *db/db* T2D mice (n = 5-6/ group). Positive correlations are represented in red and negative correlations in blue. Darkness of color and large circle size indicate a strong correlation. Factors are sorted by clusters and factors with strong positive correlations grouped to the same cluster. Significance was set at $p < 0.05$. Insignificant correlations are blank.

Disease Models & Mechanisms • Supplementary information

Table S1. Glom 5x5 SOM gene Module Assignment

Click here to download Table S1

Table S2. Glom Cluster 1 Genes

Click here to download Table S2

Table S3. Glom Cluster 2 Genes

Click here to download Table S3

Table S4. Glom Cluster 3 Genes

Click here to download Table S4

Table S5. Glom Cluster 1 GO Enrichment Clustered

Click here to download Table S5

Table S6. Glom Cluster 2 GO Enrichment Clustered

Click here to download Table S6

Table S7. Glom Cluster 1 KEGG

Click here to download Table S7

Table S8. Glom Cluster 2 KEGG

Click here to download Table S8

Table S9. Glom Cluster 3 GO Enrichment Clustered

Click here to download Table S9

Table S10. Glom Cluster 3 KEGG

Click here to download Table S10

Table S11. SCN 5x5 SOM gene Module Assignment

Click here to download Table S11

Table S12. SCN Cluster 1 Genes

Click here to download Table S12

Table S13. SCN Cluster 2 Genes

Click here to download Table S13

Table S14. SCN Cluster 3 Genes

Click here to download Table S14

Table S15. SCN Cluster 4 Genes

Click here to download Table S15

Table S16. SCN Cluster 5 Genes

Click here to download Table S16

Table S17. SCN Cluster 6 Genes

Click here to download Table S17

Table S18. SCN Cluster 1 Genes GO enrichment

Click here to download Table S18

Disease Models & Mechanisms • Supplementary information

Table S19. SCN Cluster 4 GO Enrichment Clustered

Click here to download Table S19

Table S20. SCN Cluster 5 GO Enrichment Clustered

Click here to download Table S20

Table S21. SCN Cluster 2 GO Enrichment Clustered

Click here to download Table S21

Table S22. SCN Cluster 3 GO Enrichment Clustered

Click here to download Table S22

Table S23. SCN Cluster 6 GO Enrichment Clustered

Click here to download Table S23

Table S24. SCN Cluster 3 KEGG

Click here to download Table S24

Table S25. SCN Cluster 6 KEGG

Click here to download Table S25

Table S26. SCN Cluster 2 KEGG

Click here to download Table S26

Table S27. SCN cont vs SCN STZ DEGs

Click here to download Table S27

Table S28. SCN cont vs SCN dbdb DEGs

Click here to download Table S28

Table S29. Glom cont vs Glom STZ DEGs

Click here to download Table S29

Table S30. Glom cont vs Glom dbdb DEGs

Click here to download Table S30

Table S31. Glom STZ unique DEGs

Click here to download Table S31

Table S32. Glom dbdb unique DEGs

Click here to download Table S32

Table S33. Glom common tissue DEGs

Click here to download Table S33

Table S34. SCN STZ unique DEGs

Click here to download Table S34

Table S35. SCN dbdb unique DEGs

Click here to download Table S35

Table S36. SCN common tissue DEGs

Click here to download Table S36

Table S37. Glom common tissue DEG GO

Click here to download Table S37

Table S38. Glom common tissue DEG KEGG

Click here to download Table S38

Table S39. Glom STZ unique DEG GO

Click here to download Table S39

Table S40. Glom dbdb unique DEG GO

Click here to download Table S40

Table S41. Glom dbdb unique DEG KEGG

Click here to download Table S41

Table S42. SCN common tissue DEG GO

Click here to download Table S42

Table S43. SCN STZ unique DEG GO

Click here to download Table S43

Table S44. SCN dbdb unique DEG GO

Click here to download Table S44

Table S45. SCN dbdb unique DEG KEGG

Click here to download Table S45

Table S46. Shared DEGs between SCN and Glom in STZ vs ctl

Click here to download Table S46

Table S47. Shared STZ DEG GO

Click here to download Table S47

Table S48. Shared STZ DEG KEGG

Click here to download Table S48

Table S49. Shared DEGs between SCN and Glom in dbdb vs ctl

Click here to download Table S49

Table S50. Shared dbdb DEG GO

Click here to download Table S50

Table S51. Shared dbdb DEG KEGG

Click here to download Table S51
